# Quantitative analysis of posture and gait in the differential diagnosis of functional and organic motor disorders: a multicenter cross-sectional study

**DOI:** 10.3389/fbioe.2026.1872332

**Published:** 2026-10-05

**Authors:** Michela Russo, Roberto Erro, Carlo Ricciardi, Marialuisa Gandolfi, Angela Sandri, Marianna Amboni, Elisabetta Sarasso, Andrea Gardoni, Silvia Basaia, Sofia Cuoco, Immacolata Carotenuto, Claudia Vinciguerra, Annibale Botto, Daniele Giuseppe Romano, Ilaria Antonella Di vico, Mirta Fiorio, Serena Camozzi, Giulia Pedrotti, Anna Fratucello, Giancarlo Mansueto, Francesca Benedetta Pizzini, Marco Barillari, Matteo Francesco Lauriola, Mariachiara Tozzi, Francesca Rusciano, Christian Geroin, Melania Fasoli, Angela Marotta, Francesca Salaorni, Giovanna Maddalena Squintani, Sara Mariotto, Stefano Tamburin, Fabio Paio, Giuseppe De Biasi, Giuseppe Piscosquito, Elisa Sibilla, Elisa Canu, Massimo Filippi, Federica Agosta, Maria Romano, Paolo Barone, Francesco Amato, Michele Tinazzi, Maria Teresa Pellecchia

**Affiliations:** 1 Department of Chemical, Material and Industrial Production Engineering, University of Naples Federico II, Naples, Italy; 2 Neurology Unit, University Hospital “San Giovanni di Dio e Ruggi D’Aragona”, Salerno, Italy; 3 Department of Medicine, Surgery and Dentistry, “Scuola Medica Salernitana”, University of Salerno, Baronissi, Italy; 4 IRCCS SYNLAB SDN, Naples, Italy; 5 Department of Electrical Engineering and Information Technologies, University of Naples Federico II, Naples, Italy; 6 Department of Neurosciences, Biomedicine and Movement Sciences, University of Verona, Verona, Italy; 7 Azienda Ospedaliera Universitaria Integrata Verona, Verona, Italy; 8 Neuroimaging Research Unit, Division of Neuroscience, IRCCS San Raffaele Scientific Institute, Milan, Italy; 9 Neurotech Hub, Vita-Salute San Raffaele University, Milan, Italy; 10 Department of Neuroscience, Rehabilitation, Ophthalmology, Genetics and Maternal Child Health, University of Genoa, Genoa, Italy; 11 UOC Neuroradiologia Diagnostica Ed Interventistica Azienda Ospedaliera-Universitaria “San Giovanni di Dio e Ruggi D’Aragona”, Salerno, Italy; 12 Department of Diagnostic and Public Health, Section of Radiology, University of Verona, Verona, Italy; 13 Department of Radiology, Ospedale G.B. Rossi AOUI Verona, Verona, Italy; 14 Neurorehabilitation Unit, IRCCS San Raffaele Scientific Institute, Milan, Italy; 15 Department of Surgery, Dentistry, Pediatrics, and Gynecology, University of Verona, Verona, Italy; 16 Neurology Unit, IRCCS San Raffaele Scientific Institute, Milan, Italy; 17 Neurophysiology Service, IRCCS San Raffaele Scientific Institute, Milan, Italy; 18 Vita-Salute San Raffaele University, Milan, Italy

**Keywords:** functional motor disorders, gait analysis, organic motor disorders, postural control, quantitative assessment

## Abstract

**Background:**

Functional Motor Disorders (FMDs) are characterized by impaired motor control in the absence of identifiable structural abnormalities, making their distinction from Organic Motor Disorders (OMDs) a persistent clinical challenge. Quantitative evaluation of gait and postural control may offer objective markers to characterize motor abnormalities and support the differential diagnosis between functional and organic conditions. This study aimed to characterize gait and postural performance in individuals with FMDs compared to those with OMDs and healthy controls (HCs), using dual-task and sensory manipulation paradigms, and to examine their relationship with psychometric variables.

**Methods:**

In this multicenter cross-sectional study, patients with clinically definite FMDs (n = 149), OMDs (n = 99), and HCs (n = 153) underwent standardized assessments of posture and gait under single- and dual-task conditions (both motor and cognitive), as well as with eyes open and closed. Spatiotemporal gait parameters, stabilometric measures, and dual-task effects were analyzed. Psychometric variables were also collected to explore their association with motor performance.

**Results:**

Compared to OMDs and HCs, individuals with FMDs exhibited significantly greater postural instability, an opposite response to dual-task conditions, and increased stride time variability (all p < 0.05). In this group, motor performance was significantly associated with anxiety, depression, and fatigue, whereas alexithymia was associated with poorer gait performance only in OMDs and HCs (p < 0.05).

**Discussion:**

In conclusion, FMDs display a distinct motor and behavioral profile, particularly in response to dual-task paradigms. Quantitative measures such as dual-task effect, sway area under eyes-closed conditions, and stride time variability (all p < 0.05) may represent promising markers for differentiating functional from organic motor disorders.

## 1 Introduction

Functional motor disorders (FMDs) are a group of highly debilitating neurological conditions that belong to the broader spectrum of functional neurological disorders (non-epileptic seizures, somatosensory and visual disturbances, and pain syndromes) (Baizabal-Carvallo et al., 2020).

Functional neurological disorders have an estimated incidence of 4–12 per 100,000 per year and a prevalence of approximately 50 per 100,000 in neurological settings. Among these, FMDs represent the most common subtype, accounting for more than 50% of cases and up to 15%–20% of patients attending neurology clinics (“Epidemiology,” 2016; Espay et al., 2018).

Clinically, FMDs cause highly disabling motor symptoms that may involve both deficits of movement production, such as weakness and slowness, and excessive movements, such as tremors and dystonia (Tinazzi et al., 2020; 2021b; Serranová et al., 2023). These symptoms may occur in isolation or combination and are frequently associated with other functional symptoms, as well as non-motor manifestations, including fatigue and pain, contributing to reduced functional independence and poor quality of life (Tinazzi et al., 2020; Serranová et al., 2023; Gandolfi et al., 2021b).

Functional gait disorders represent one of the most frequent and disabling manifestations of FMDs (Tinazzi et al., 2021b; Nonnekes et al., 2020). They are present in 23%–45% of patients with the condition, either alone or alongside other motor and non-motor symptoms (“Epidemiology,” 2016). These disturbances are characterized by a wide range of gait patterns, including slow and cautious walking, astasia–abasia, knee buckling, or “tightrope-like” gait, often accompanied by excessive effort and instability in the absence of objective balance deficits (Espay et al., 2018; Nonnekes et al., 2020). These disorders are typically defined by inconsistency and incongruence with known neurological gait patterns, and marked variability over time and across contexts (Nonnekes et al., 2020). Inconsistency refers to the variability of symptoms over time and across different attentional contexts (Espay et al., 2018). For instance, symptoms of severity, frequency, or movement pattern can change with distraction or worsen with focused attention on the body. Incongruence refers to the mismatch between the clinically observed motor pattern and those typically associated with known neurological disorders, also known as organic movement disorders (OMDs) (Espay et al., 2018). For instance, incongruence includes movements that are recognized as neuroanatomical or pathophysiological, possibly with an organic lesion of the Central Nervous System. In contrast to OMDs, which result from identifiable neurological damage, as in Parkinson’s disease (PD) and other neurodegenerative conditions [8], FMDs did not. Together, inconsistency and incongruence reflect altered motor control mechanisms and are considered key clinical features supporting the diagnosis of FMDs and exclude organic disorders (Espay et al., 2018).

Currently, gait and postural control assessment in FMDs relies largely on neurological examination, including the evaluation of positive clinical signs such as inconsistency and incongruence through bedside gait tests (e.g., straight walking, dual-tasking, backward walking, and walking with eyes closed), together with disease-specific rating scales such as the Simplified Functional Movement Disorders Rating Scale (S-FMDRS), all of which remain partially dependent on subjective interpretation and potential bias (Nielsen et al., 2017; Nonnekes et al., 2020; Serranová et al., 2023). In contrast, a quantitative evaluation using tools such as posturography and gait analysis provides a more in-depth understanding of postural and gait mechanisms. This approach enables exploring the specific features of motor performance and supports the development of personalized rehabilitation strategies aimed at improving quality of life.

Postural control and walking are complex functions that depend on sensorimotor, cognitive, visuospatial, and motor planning processes (Woollacott and Shumway-Cook, 2002). From a physiological perspective, balance ranges from automatic, low-level adjustments to higher-level, cognitively controlled responses (Boisgontier et al., 2013). Emotional and psychological factors, including anxiety and fear of falling, may further increase conscious control of posture, thereby interfering with automatic motor regulation (Huffman et al., 2009). Studies using posturography in older adults and patients with neurological deficits have emphasized the important role of attentional processes in maintaining stability (de Souza Fortaleza et al., 2017; Russo et al., 2022; Pisani et al., 2024; Bekkers et al., 2018).

In this context, previous studies have shown that gait abnormalities may improve under distraction or dual-task conditions, suggesting that excessive self-focused attention plays a key role in disrupting automatic motor control (Nonnekes et al., 2020; Di Filippo et al., 2025; Russo et al., 2025; 2026). Moreover, quantitative analyses have highlighted increased variability and altered spatiotemporal parameters, further supporting the hypothesis of impaired integration between cognitive and sensorimotor processes (Gandolfi et al., 2023). Consistently, recent evidence indicates that gait in functional gait disorders is strongly influenced by attentional demands, with dual-task conditions modulating spatiotemporal performance and revealing altered high-level motor control mechanisms (Sandri et al., 2025).

The pathophysiology of FMDs remains incompletely understood. According to emerging models of predictive coding, symptoms can be conceptualized as aberrant perceptual inferences about the body’s state. Neural systems actively integrate interoceptive and exteroceptive information to generate predictions that guide motor and behavioral responses. Disturbances in these processes may lead to dysfunctional integration between sensory inputs and motor control, ultimately contributing to symptom generation and disability (Hallett et al., 2022).

It has been proposed that different functional motor symptoms may share a common underlying pathophysiological mechanism (Hallett et al., 2022). Despite the wide heterogeneity of clinical manifestations, including weakness, tremor, dystonia, and gait disturbances, current models suggest that all these symptoms may arise from similar alterations in the integration between sensory predictions, attention, and motor control processes. However, it remains unclear why a given patient develops a specific motor phenotype rather than another. This variability is likely influenced by complex interactions among individual factors, including prior experiences, cognitive and emotional processes, and context-dependent modulation, which may shape symptom expression within a shared neurobiological framework (Hallett et al., 2022; Espay et al., 2018).

Most studies have focused either on clinical phenomenology or on isolated aspects of gait performance; little attention has been focused on static postural control disturbances, and only a limited number have combined quantitative gait analysis with postural control assessment within a unified experimental framework. More recently, multimodal approaches have started to address these limitations. In particular, Gandolfi et al. (2026) combined behavioral, motor, neurophysiological, and imaging measures within an integrated framework, identifying altered multisystem integration in FMDs and suggesting potential diagnostic biomarkers. However, direct comparisons between FMDs and OMDs remain scarce, limiting the identification of condition-specific motor signatures.

To address these knowledge gaps, the present study was conducted within a larger multicentre project aimed at identifying diagnostic and prognostic biomarkers in Functional Motor Disorders (Gandolfi et al., 2024). Specifically, this study focused on the quantitative characterization of gait and postural control in patients with FMDs compared with OMDs and HCs, using a standardized experimental paradigm combining dual-task conditions and sensory manipulation (eyes open vs. eyes closed). We aimed to identify objective instrumental features that may serve as potential biomarkers to support the differential diagnosis between FMDs and OMDs. Additionally, we aimed to investigate the relationship between motor performance and psychometric variables to better characterize the factors underlying postural and gait dysfunction in FMDs.

This evaluation was conducted in a broad, representative cohort of patients with a clinically definite diagnosis of FMDs, including a wide range of motor symptoms and phenotypes, to capture shared underlying mechanisms across different clinical presentations (Gandolfi et al., 2024).

We hypothesized that patients with FMDs would exhibit distinct patterns of gait and postural control in response to attentional load and sensory manipulation, reflecting altered integration of sensory inputs with higher-level control processes. In contrast, patients with OMDs and HCs would show an opposite or more physiologically consistent modulation of performance.

## 2 Materials and methods

### 2.1 Study design and participants

The multicenter cross-sectional study represents a predefined secondary analysis of the larger prospective project described by Gandolfi et al. (2024). The study is reported in accordance with the Strengthening the Reporting of Observational Studies in Epidemiology (STROBE) statement (Supplementary Material, STROBE guidelines). The study included 401 subjects, divided into three groups: healthy controls (HCs, n = 153), patients with a clinically definite diagnosis of FMD (FMDs, n = 149) (Thenganatt and Jankovic, 2019) and patients with organic motor disorders (OMDs, n = 99).

Among FMDs, 36 presented with an isolated motor symptom (including tremor, weakness, dystonia, and gait impairment), while the remaining patients showed combined phenotype presentations. The phenotypic distribution of the FMDs cohort is reported in Supplementary Table S1. Among OMDs, 44 were essential tremors, 52 had dystonia, and four had myopathies.

The inclusion of different OMDs phenotypes was intended to better match the clinical heterogeneity of the FMD cohort. Since FMDs encompass a broad spectrum of motor manifestations (e.g., weakness, tremor, dystonia, and gait disorders), our objective was not to compare individual disease entities but rather to identify quantitative motor features that distinguish functional from OMDs as broad diagnostic categories encountered in clinical practice. This approach is consistent with current pathophysiological models suggesting that different FMD phenotypes may share common alterations in sensorimotor integration, attentional control, and motor processing (Hallett et al., 2022). Accordingly, the inclusion of different OMDs phenotypes provided a clinically relevant comparator reflecting the heterogeneity of neurological disorders commonly considered in the differential diagnosis of FMDs. This design might reproduce the real-world clinical scenario in which neurologists are required to differentiate FMDs from a broad spectrum of organic motor disorders with overlapping motor manifestations, rather than from a single neurological condition.

Participants were recruited at the Neurological Unit B of the AOUI of Verona (Verona, Italy) and at the Neurological Clinic of the AOU San Giovanni di Dio e Ruggi d’Aragona (Salerno, Italy) between March 2023 and November 2024 (Gandolfi et al., 2024).

For all groups, the inclusion criteria were age 18 or older and the ability to provide written informed consent. For the OMDs group, patients were included if diagnosed with one of the following conditions: essential tremor (ET), according to the 2018 consensus criteria (Bhatia et al., 2018); idiopathic adult -onset dystonia (focal, segmental, or multifocal), diagnosed according to established criteria (Albanese et al., 2013) and patients with muscle diseases including idiopathic inflammatory myopathies, sporadic inclusion body myositis and limb-girdle muscular dystrophies were diagnosed according to published criteria (Engel et al., 2004). All OMDs diagnoses were confirmed by movement disorder specialists.

Exclusion criteria for all participants were: Mini-Mental State Examination score lower than or equal to 24/30; inability to comply with the experimental protocol, inability to provide informed consent for participation; certified neurological and/or psychiatric comorbidities (i.e., neuropathy, seizures, major depression), severe disease interfering with deambulation, and contraindications for 3T MRI.

The participant selection process, including eligibility assessment, application of inclusion and exclusion criteria, and the final sample composition, is summarized in Figure 1.

**FIGURE 1:**
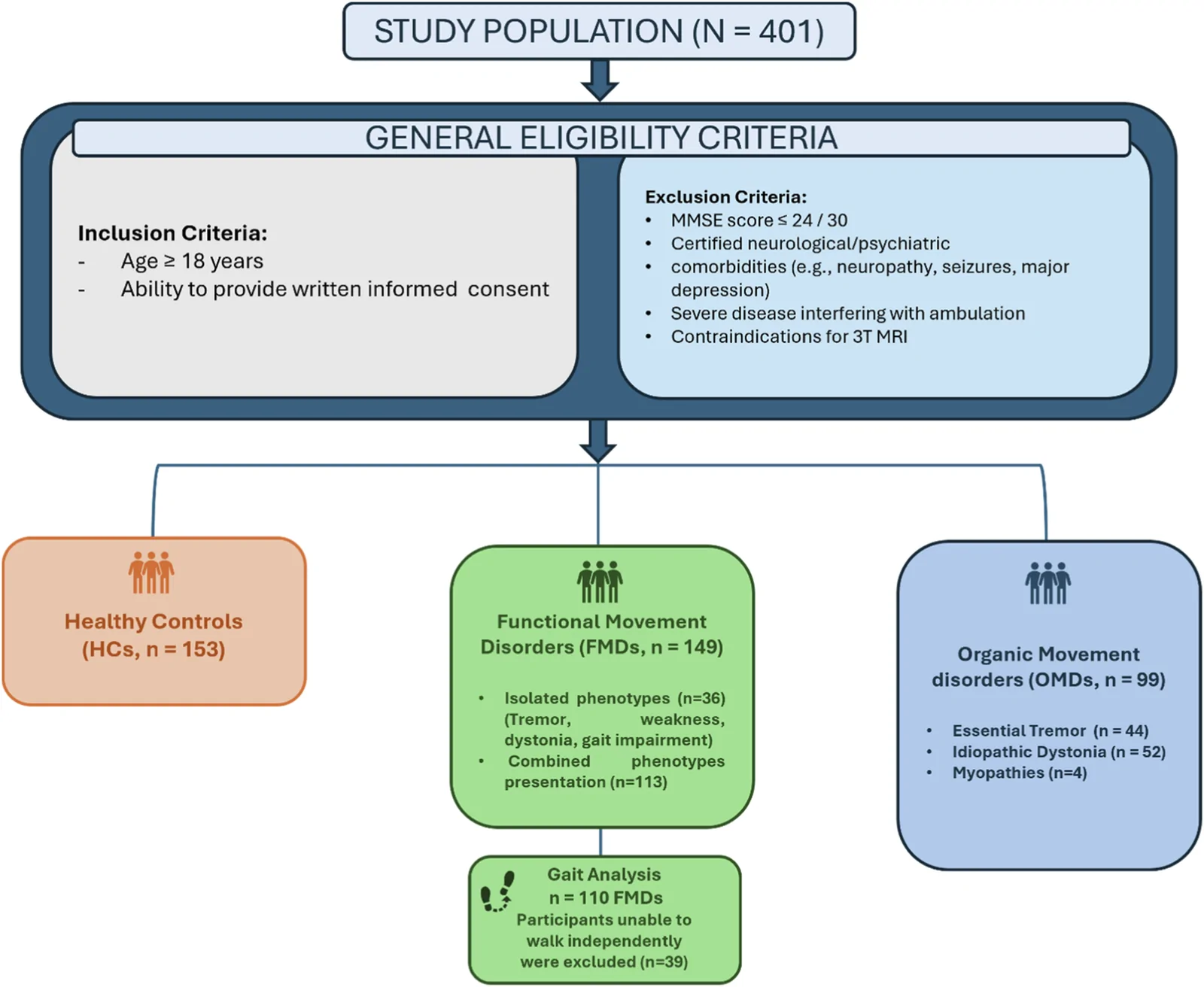
Flow diagram illustrating participant selection, inclusion and exclusion criteria, and final sample size across the three study groups.

Ethics approval was obtained from the respective committees (4201CESC - PNRR-MAD-2022–12376826). The trial was registered in clinicaltrials.gov (NCT06328790). All patients provided written informed consent. The study adhered to the ethical standards for research involving human participants as outlined in the Declaration of Helsinki.

### 2.2 Clinical assessment and psychometric assessment

Demographic and clinical data were prospectively collected in accordance with the study protocol (Gandolfi et al., 2024), in compliance with the General Data Protection Regulation. Motor symptom severity in patients with FMDs was assessed using the objective-rated S-FMDRS (range 0–54), with higher scores indicating greater motor symptom severity (Nielsen et al., 2017). Psychometric measures were included to characterize non-motor dimensions that may influence motor control and postural regulation. In particular, alexithymia (TAS-20), anxiety and depressive symptoms (BAI, BDI) (Beck et al., 2012; Upton, 2013), fatigue (MFI) (Smets et al., 1995), and body awareness (BAQ) were assessed as proxies of cognitive, emotional, and interoceptive processes. These factors are known to modulate attentional allocation, sensorimotor integration, and motor variability, and may therefore contribute to the observed alterations in postural and gait control. Finally, the perception of health-related quality of life was investigated using the Short Form-12 Health Survey (SF-12) - Physical Component Score (SF12-PCS) and Mental Component Score (SF12-MCS) (Ware et al., 1996).

### 2.3 Instrumental postural control and gait assessment

#### 2.3.1 Postural control assessment

Stabilometric analysis was employed to assess static postural control using a force platform (Prokin 252, Tecnobody™, Italy; https://www.tecnobody.com/prokin; Üzümcü et al., 2024), a highly sensitive instrument for recording center of pressure (CoP) trajectories during quiet standing. To ensure reproducibility, the subject’s posture was standardized using a special V-shaped frame, which maintained a constant distance of 3 cm between the medial malleoli and an external rotation of the feet of 12° with respect to the anterior-posterior axis. During the test, the subjects stood on the platform’s solid surface without support from their upper limbs.

Each participant performed stabilometric trials under single–task and dual–task conditions, both with eyes open (EO) and eyes closed (EC) (See Figure 2). Each trial lasted 30 s.

**FIGURE 2:**
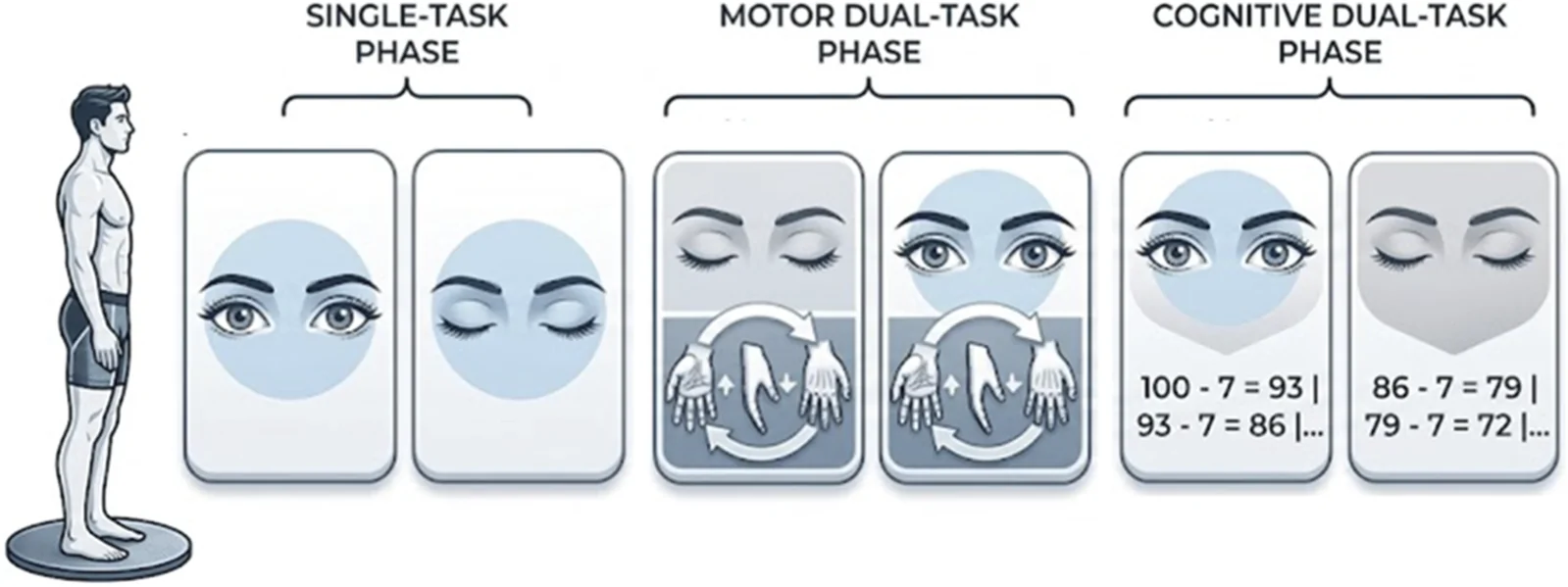
Experimental setup for stabilometric assessment.

The Single Task (ST) condition served as a baseline control, in which the subject was instructed to maintain an upright position while staring at a reference cross in the center of a screen in front of them, without performing any concurrent tasks.

Dual-task conditions consisted of motor and cognitive dual-tasking. During the motor Dual-Task (mDT), an interfering motor task was added, consisting of pronation-supination movement of the dominant hand while keeping fixation on the visual target on the screen (Gandolfi et al., 2021a). During the cognitive Dual-Task (cDT), a serial subtraction of the number 7, starting from a random numerical value provided by the operator, was performed while maintaining fixation on the visual target on the screen (Gandolfi et al., 2021a).

The following stabilometric parameters of interest were extracted: sway area (mm2), which is represented by the area covered by CoP displacement; sway path (perimeter) (mm), which is the total length of the CoP trajectory; and the Romberg index, which is the ratio between sway area in EC and OC conditions. The selected stabilometric parameters were chosen based on the literature on FMDs (Gandolfi et al., 2021a). The comparison between EO and EC conditions allows assessment of sensory reweighting mechanisms and the degree of visual dependency required to maintain static postural control (Peterka, 2002). In addition, in patients with FMDs, the lack of visual feedback may further increase the internally directed attentional focus toward body sensations (Edwards et al., 2012). Therefore, this condition interferes with automatic motor control in FMDs by increasing excessive self-focused attention and then investigating the integration between sensory and attentional mechanisms underlying postural control (Edwards et al., 2012).

#### 2.3.2 Gait analysis

Gait analysis was performed using wearable instrumented insoles (FeetMe® Insoles, France; https://feetme.fr/en; Farid et al., 2021), integrating inertial measurement unit (IMU) sensors for the continuous recording of spatiotemporal gait parameters during walking. Participants were instructed to wear comfortable, self-selected footwear and to perform the task without any assistive devices. All assessments were conducted under standardized conditions, including consistent instructions and walking distance. Each participant was instructed to walk along a straight 10-m corridor at a self-selected pace under both single-task conditions (i.e., simple walking) and different dual-task conditions. The motor dual-task (mDT) consisted of repeatedly performing pronation and supination movements (i.e., rotating the palm up and down) with the dominant hand while walking; the cognitive dual-task (cDT) required participants to perform serial subtractions starting from 100 and subtracting seven repeatedly (e.g., 100–7, 93–7, 86–7, etc.); the visual dual-task (vDT) involved focusing on a fixed circular target positioned at eye level on the wall at the end of the 10-m corridor while walking, thereby imposing an additional visual attentional demand during gait and engaging visuo-motor integration. All assessments were performed in an outpatient clinical setting, ensuring that participants could perform the walking tasks under comfortable and adequate visual and thermal conditions. A schematic representation of the experimental setup is provided in Figure 3.

**FIGURE 3:**
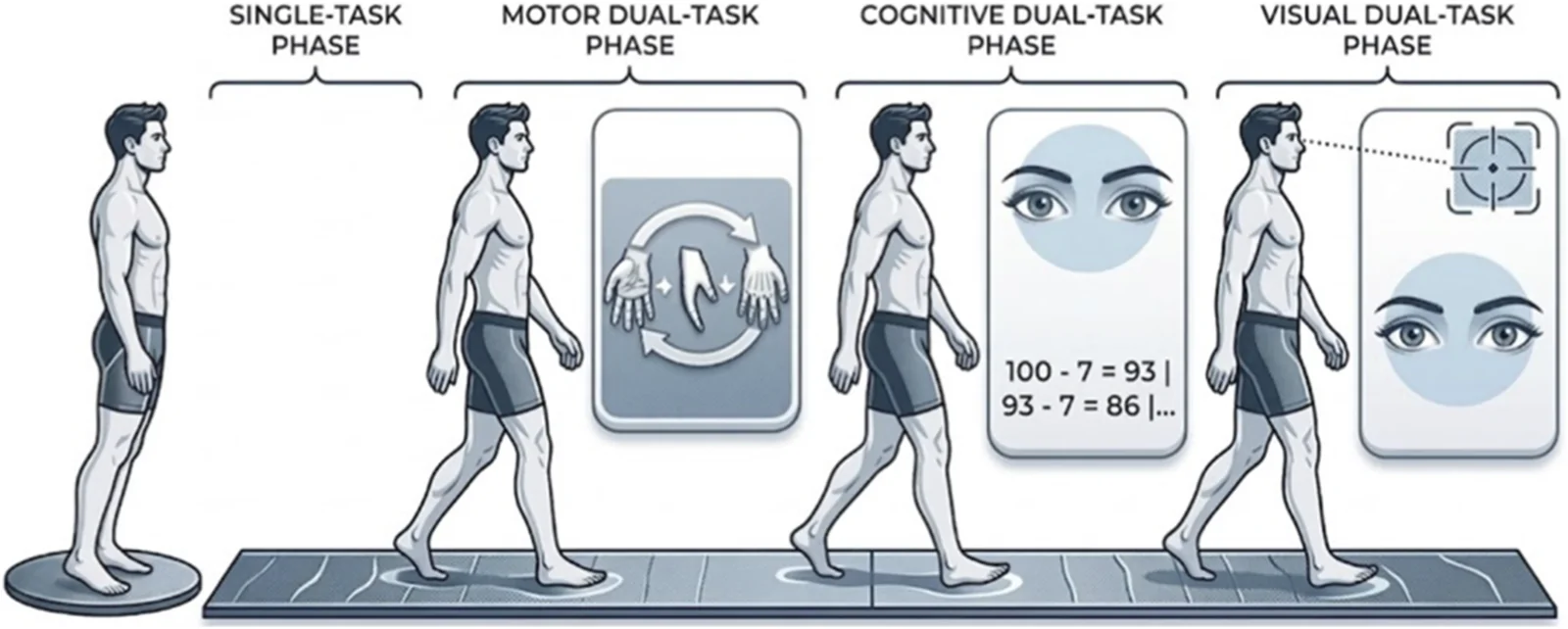
Experimental conditions involved in the gait analysis.

#### 2.3.3 Dual-task effect (DTE)

The Dual-Task Effect (DTE) was calculated to quantify the effect of dual-tasking as the relative change between dual -task and single–task conditions according to the following formula:

$$DTE\left(\%\right)=\frac{Dual\;Task\;Performance-Single\;Task\;Performance}{Single\;Task\;Performance}\times 100$$

Based on this approach, since higher values of spatio-temporal parameters (i.e., speed or step length) indicate better performance, a positive DTE reflects an improvement in performance during the dual-task. In contrast, a negative DTE indicates worsening and represents a “cost” induced by interference from the secondary task. In contrast, for parameters for which higher numerical values correspond to decreased performance (i.e., step variability, the sway area, or the CoP path perimeter), the interpretation of the DTE sign is reversed.

### 2.4 Statistical analysis

No formal priori sample size calculation was performed for this predefined secondary analysis, as the study population was determined by the main multicentre project described by Gandolfi et al. (2024).

Demographic and clinical variables were assessed using descriptive and inferential statistics.

Instrumental postural control and gait parameters were analyzed separately using Linear Mixed-Effects Models (LMMs). LMMs were selected to account for the repeated-measures design and the correlation among observations collected from the same participant under different conditions. Group (FMDs, OMDs, and HC), condition (single-task and dual-task), and their interaction were included as fixed effects. Subject was included as a random effect to account for inter-individual variability and repeated measurements within participants. Age, sex, and height were included as covariates (fixed effects) to control for their potential influence on performance. This approach enabled the evaluation of group differences, condition effects, and Group × Condition interactions while adjusting for relevant demographic and anthropometric characteristics. The Group effect tested overall differences between HC, FMD, and OMD participants across all conditions, while the Task effect evaluated differences between single-task and dual-task conditions irrespective of group membership. The Group × Task interaction examined whether the effect of task condition differed across diagnostic groups. The assumptions of the LMMs, including normality of residuals and homoscedasticity, were verified and supported the adequacy of the models. Missing data were handled within the LMM framework assuming a missing-at-random mechanism. When significant main effects or interactions were observed, *post hoc* pairwise comparisons were performed using estimated marginal means. Bonferroni correction was applied to adjust for multiple comparisons. For pairwise comparisons, effect sizes were additionally calculated using Cohen’s d to quantify the magnitude of between-group differences. Cohen’s d values were interpreted according to conventional thresholds, with values of 0.2, 0.5, and 0.8 indicating small, medium, and large effects, respectively. For the fixed effects derived from the LMMs, partial eta squared (ηp^2^) was reported as an additional measure of effect size. According to Cohen’s conventions, ηp^2^ values of 0.01, 0.06, and 0.14 were considered indicative of small, medium, and large effects, respectively (Cohen J. et al., 1988; Richardson, 2011).

#### 2.4.1 Exploratory subgroup analyses

To further explore the potential influence of clinical heterogeneity within the diagnostic groups, we additionally performed exploratory phenotypic comparisons within the FMD and OMD cohorts. Specifically, within the FMD cohort, patients were stratified according to their presenting motor manifestations, including patients with isolated single-symptom presentations, to explore whether different clinical manifestations were associated with differences in postural control parameters. Within the OMD cohort, subgroup comparisons were performed according to the main clinical manifestations (i.e., tremor and dystonia). Specifically, differences among the clinical phenotypes included in each diagnostic category were assessed using parametric and non-parametric comparisons (ANOVA or Kruskal–Wallis test) according to data distribution, followed by appropriate *post hoc* analyses when applicable.

#### 2.4.2 Sensitivity analyses

To evaluate the robustness of the primary between-group findings with respect to the clinical heterogeneity of the included populations, additional sensitivity analyses were performed. Specifically, the primary between-group comparisons were repeated using more homogeneous clinical subgroups within the OMD cohort (essential tremor and dystonia analyzed separately). For the OMD cohort, the main analyses were repeated separately considering patients presenting tremors and patients presenting dystonia, including comparisons among HCs, FMDs, and OMDs with tremor manifestations, and among HCs, FMDs, and OMDs with dystonia manifestations. Furthermore, a phenotype-matched sensitivity analysis was performed by comparing HCs, OMDs patients with tremor, and FMDs patients presenting tremor without concomitant dystonia, to assess whether the observed group differences remained consistent within clinically comparable tremor phenotypes. Given the mixed clinical presentation of FMD, the tremor subgroup was defined as patients presenting tremors as a clinical manifestation without concomitant dystonia, while other motor manifestations could coexist.

These analyses were performed to assess whether the magnitude and direction of the primary between-group differences remained consistent across clinically more homogeneous subgroups.

Finally, Pearson or Spearman correlation analyses (depending on data distribution) were performed as exploratory analysis to investigate potential relationship between psychometric measures and instrumental motor parameters, in order to evaluate the relationships between clinical/psychological characteristics and objective measures of postural control and gait performance. For correlation analyses, false discovery rate (FDR) correction using the Benjamini–Hochberg procedure was applied separately within each experimental group. An adjusted p-value <0.05 was considered statistically significant.

Potential sources of confounding were addressed by including age, sex, and height as covariates.

All statistical analyses were conducted in SPSS (v.30).

## 3 Results

The results section is organized as follows. First, the clinical and demographic characteristics of the participants are presented. Subsequently, the findings of the postural control and gait analyses are reported. Finally, psychometric measures are described, and their associations with postural control and gait parameters are examined through correlation analyses.

### 3.1 Clinical and demographical characteristics

The study population included 149 participants with FMDs, who were compared with 99 participants with OMDs and 153 HCs. Demographic and anthropometric characteristics are reported in Table 1. Participants with FMDs had a mean age of 44.78 ± 13.90 years, which was comparable to that of HCs (43.52 ± 13.43 years) and lower than that of OMDs (65.02 ± 9.10 years), resulting in a significant group effect (p < 0.001; Table 1). The FMDs group included 114 females and 36 males, showing a higher proportion of females than the OMDs group (53 females and 46 males; p < 0.001; Table 1), whereas gender distribution was more similar to that observed in HCs (97 females and 56 males). Participants with FMDs were also significantly shorter in height than the comparison groups, likely reflecting the higher proportion of females in this cohort. Educational level differed across groups, with HCs being more highly educated than both patient groups. Given these demographic and anthropometric differences, age, gender, and height were included as covariates in subsequent analyses.

**TABLE 1:** Results of the statistical analysis for the clinical-demographic variables across the three experimental groups (HC, FMD, OMD). Data are expressed as mean ± standard deviation (SD). Effect sizes (Cohen’s d) are reported for statistically significant group pairwise comparisons. The level of statistical significance was set at p < 0.05; *post hoc* comparisons were corrected using the Bonferroni method.

Variables	1.HC (n = 153)	2.FMD (n = 149)	3.OMD (n = 99)	p-value	Cohen’s d
Gender	F: 97; M: 56	F: 114; M: 36	F: 53; M: 46	**<0.05 (2–3)**	​
Age (years)	43.52 ± 13.43	44.78 ± 13.90	65.02 ± 9.21	**<0.001 (1–3; 2–3)**	d_1-3_ = −1.80; d_2-3_ = -1.65
Height	1.70 ± 0.09	1.65 ± 0.08	1.69 ± 0.10	**<0.001 (1–2)**	d_1-2_ = 0.59
Weight	68.40 ± 10.61	68.97 ± 16.07	73.22 ± 14.70	0.647	​
BMI	23.43 ± 2.59	24.45 ± 4.48	25.30 ± 3.57	**0.014 (1–3)**	d_1-3_ = −0.62
Education	15.92 ± 2.66	12.28 ± 3.60	10.95 ± 4.47	**<0.001 (1–3; 1–2)**	d_1-3_ = 1.43; d_1-2_ = -1.15

F: female; M: male; BMI: Body Mass Index. According to Cohen’s conventions, d values of 0.2, 0.5, and 0.8 indicate small, medium, and large effects, respectively.

Bold p values indicate statistical significance (p < 0.05).

### 3.2 Postural control assessment

Patients with FMDs exhibited significantly poorer postural control than both HCs and OMDs across all experimental conditions (see Table 2). Specifically, FMDs showed significantly higher mean sway area and perimeter in both EO and EC conditions across all postural tasks. These differences were confirmed by the mixed-effects model, which revealed a highly significant main effect of group (p < 0.001; Table 2) across all included variables.

**TABLE 2:** Results of the statistical analysis for the stabile parameters across the three experimental groups (HCs, FMDs, OMDs). Data are expressed as mean ± standard deviation. The level of statistical significance was set at p < 0.05; *post hoc* comparisons were corrected using the Bonferroni method.

Variables	Group	ST	mDT	cDT	Group effect p-value	Task effect p-value	T x G
EC sway area	1.HC 2.FMD 3.OMD	166.19 ± 84.61 447.13 ± 405.27 211.01 ± 120.78	211.45 ± 92.18 461.53 ± 393.40 261.89 ± 146.95	224.95 ± 157.96 498.26 ± 389.04 313.50 ± 158.01	**<0.001 (1-2; 2-3);** ηp ² = 0.321	**<0.001** (ST-mDT)* ;**0.029** (ST-mDT)*	0.624
EC sway perimeter	1.HC 2.FMD 3.OMD	257.59 ± 63.99 412.43 ± 235.99 328.95 ± 83.30	466.90 ± 165.81 686.04 ± 438.26 439.59 ± 147.90	326.31 ± 91.91 455.32 ± 198.25 450.79 ± 147.24	**<0.001 (1-2; 2-3),** **0.024 (1-3);** ηp² = 0.222	**<0.001** (ST-mDT*; mDT-cDT^A,B^; ST-cDT*)	<0.001*
EC sway area	1.HC 2.FMD 3.OMD	348.58 ± 226.25 1353.25 ± 1261.73 467.54 ± 285.34	398.55 ± 193.70 1009.96 ± 927.04 479.98 ± 299.97	285.43 ± 159.22 776.46 ± 702.68 443.42 ± 312.70	**<0.001 (1-2; 2-3);** ηp² = 0.310	**<0.001** (ST-cDT^A,B^; **0.048** DT-mDT ^A,B^)	**<0.001** ^**B,C**^
EC sway perimeter	1.HC 2.FMD 3.OMD	444.98 ± 158.55 753.86 ± 449.04 594.79 ± 247.85	637.40 ± 259.72 964.82 ± 560.78 671.62 ± 242.98	443.93 ± 147.24 642.85 ± 344.99 593.99 ± 223.17	**<0.001 (1-2; 2-3);** ηp² **=** 0.206	**<0.001** (ST-mDT*; mDT-cDT*)	**<0.001** ^**A,B**^
Romberg index (%)	1.HC 2.FMD 3.OMD	205.36± 91.00 290.01± 166.39 224.57 ± 109.66	174.48 ± 81.59 199.34 ± 96.94 199.56 ± 102.24	142.65 ± 92.73 151.54 ± 80.66 142.60 ± 65.68	**<0.001 (1-2), 0.020 (2-3);** ηp² = 0.060	**<0.001** (ST-mDT*; mDT-cDT*; ST-cDT*)	<0.001*

Oe: Open-eyes; CE: close-eyes; ST: Single task; mDT: motor dual-task; cDT: Cognitive dual-task; T: Task, G; Group; HC: Healthy control; FMD: Function motor disorders; OMD: Organic motor disorders. Bold *p*-values indicate statistical significance (*p* < 0.05). Superscripts applied to *post hoc* comparisons indicate in which groups the variation between tasks was significant: * indicates a significant difference observed in all groups; ^A^ indicates a significant difference only in the HC group; ^B^ indicates a significant difference only in the FMD group; ^C^ indicates a significant difference only in the OMD group. ηp^2^ indicates partial eta squared, reported as a measure of effect size for the fixed effects of the model. According to Cohen’s conventions, ηp^2^ values of 0.01, 0.06, and 0.14 correspond approximately to small, medium, and large effects, respectively

A significant main effect of group was observed for sway area in the EO condition (p < 0.001; Table 2). Pairwise comparisons confirmed significant differences between groups, with FMDs showing markedly higher sway area values than both HCs and OMDs across conditions (p < 0.001; Table 2). In addition, in the EO condition, the introduction of a dual-task (motor or cognitive) resulted in a significant deterioration of postural control in all groups (p < 0.001; pairwise analysis: ST vs. mDT; ST vs. cDT; Table 2), consistent with the expected attentional cost of dual-tasking.

Similarly, a significant main effect of group was observed for sway area in the EC condition (p < 0.001; Table 2). Pairwise analyses again revealed significant between-group differences (p < 0.001; Table 2), with FMDs showing higher sway area values compared with HCs and, to a lesser extent, OMDs. A significant main effect of task was also found (p < 0.001; Table 2), with pairwise comparisons indicating that sway area significantly increased in all groups from single-task (ST) to both motor (mDT) and cognitive dual-task (cDT) conditions (all p < 0.001; Table 2).

Under EC single-task conditions, sway area in FMDs was markedly higher than in both HCs (p < 0.001; Table 2) and OMDs (p < 0.001; Table 2). A significant task effect was also observed for all parameters (p < 0.001), with pairwise analyses confirming statistically significant differences between single-task and corresponding dual-task conditions across all populations (Table 2).

Conversely, under EC conditions, a distinctive behavioral pattern emerged in the FMD group. While both HCs and OMDs generally exhibited worsening performance during the motor dual-task and either no change or slight improvement during the cognitive dual-task (for sway area and perimeter), FMD patients showed a clear improvement in sway area in both dual-task conditions. In contrast, perimeter slightly increased during the motor dual-task and decreased during the cognitive dual-task, in line with previous literature (Gandolfi et al., 2021a).

Regarding the Romberg index, FMD patients exhibited the highest values overall, with statistically significant differences compared with both HCs (p < 0.001; Table 2) and OMDs (p = 0.021; Table 2). Both the main effect of task and the group × task interaction were significant (p < 0.001; Table 2).

Finally, the group × task (TxG) interaction was clinically relevant and statistically significant for nearly all parameters (p < 0.001), except for sway area in the EO condition (Table 2).

Regarding the DTE, sway area in the EC condition was the only variable reaching statistical significance within the FMD group (p < 0.05; Table 3). In the motor dual-task and, more prominently, in the cognitive dual-task, a clear divergence between groups was observed: HCs and OMDs showed significantly higher DTE than FMDs. Pairwise analyses revealed significant differences between HCs and FMDs (p = 0.018; Table 3) and between FMDs and OMDs (p = 0.014; Table 3). FMD patients exhibited reduced DTE values in both dual-task conditions, with negative values observed in the cognitive dual-task, whereas OMDs and HCs showed increased sway area during dual-task conditions. Finally, a significant group × task interaction was found (p = 0.006; Table 3).

**TABLE 3:** Results of the statistical analysis for the DTE on stabilo parameters across the three experimental groups (HCs, FMDs, OMDs). Data are expressed as mean ± standard deviation. The level of statistical significance was set at p < 0.05; *post hoc* comparisons were corrected using the Bonferroni method.

Variables	Group	mDT	cDT	Group Effect p-value	Task Effect p-value	T x G
DTE EO sway area	1.HC 2.FMD 3.OMD	53.42% (7.23%) 37.20% (9.63%) 45.67% (10.71%)	50.25% (8.51%) 70.46% (15.70%) 77.07% (11.15%)	0.767	**0.005** ^**B,C**^	**0.047** ^**B,C**^
DTE EO sway perimeter	1.HC 2.FMD 3.OMD	88.47% (6.06%) 83.19% (9.20%) 37.49% (5.01%)	30.06% (2.93%) 25.11% (4.70%) 39.91% (4.13%)	**0.009 (1-3)** ηp²**=** 0.034	**<0.001** ^**A,B**^	**<0.001** ^**A,B**^
DTE EC sway area	1.HC 2.FMD 3.OMD	38.68% (8.43%) 5.27% (6.64%) 19.79% (7.56%)	-2.40% (4.60%) -16.83% (5.83%) 25.03% (16.69%)	**0.014 (2-3); 0.018 (1-2)** ηp²**=** 0.038	**<0.001** ^**A,B**^	**0.006** ^**A,B,C**^
DTE EC sway perimeter	1.HC 2.FMD 3.OMD	50.62% (4.96%) 125.81% (88.73%) 23.15% (5.48%)	5.09% (2.96%) 69.15% (75.70%) 7.46% (4.03%)	0.483	<0.001*	**0.022** ^**A,B,C**^

DTE: Dual task effect; OE: open-eyes; CE: close-eyes; motor dual-task; cDT: Cognitive dual-task; T: Task, G; Group; HC: Healthy control; FMD: Function motor disorders; OMD: Organic motor disorders. Bold *p*-values indicate statistical significance (*p* < 0.05). Superscripts applied to *post hoc* comparisons indicate in which groups the variation between tasks was significant: * indicates a significant difference observed in all groups; ^A^ indicates a significant difference only in the HC group; ^B^ indicates a significant difference only in the FMD group; ^C^ indicates a significant difference only in the OMD group**.** ηp^2^ indicates partial eta squared, reported as a measure of effect size for the fixed effects of the model. According to Cohen’s conventions, ηp^2^ values of 0.01, 0.06, and 0.14 correspond approximately to small, medium, and large effects, respectively

#### 3.2.1 Exploratory subgroup analyses

Exploratory subgroup analyses did not identify statistically significant differences in postural control among the main clinical phenotypes within either the FMDs or OMDs cohorts (all p > 0.05). These findings are reported in the Supplementary Tables S2,S3.

#### 3.2.2 Sensitivity analyses

Repeating the primary analyses separately in patients with essential tremor and dystonia yielded a pattern of results that was consistent with the main analyses, with comparable magnitude and direction of the observed between-group differences (Supplementary Tables S4,S5). Statistically significant differences in postural control measures were also found when considering clinically homogeneous OMD subgroups. Specifically, significant differences were maintained across both single-task and dual-task conditions (p < 0.05), with medium-to-large effect sizes (Cohen’s d > 0.6) observed in several pairwise comparisons. Results are reported in the Supplementary Tables S4,S5.

For postural control performance compared among HCs, FMDs patients presenting tremor symptoms (including patients with additional motor manifestations but without dystonia), and OMDs patients with tremor manifestations, significant differences were maintained in the evaluated postural control measures (p < 0.05), with medium-to-large effect sizes (Cohen’s d > 0.6) observed in several pairwise comparisons. Results are reported in the Supplementary Table S6.

#### 3.2.3 Gait analysis

Gait analysis was performed in 110 out of 149 patients with FMDs. Participants were excluded from gait analysis if FMD-related motor impairment prevented them from walking independently without assistive devices or from safely completing the walking protocol. Across all analyzed conditions, HCs demonstrated significantly superior performance, characterized by longer step length, higher walking speed, and shorter gait cycle duration compared with both pathological groups (p < 0.001; pairwise analysis: HCs vs. FMDs and HCs vs. OMDs; see Table 4. In detail, a clear group effect emerged, with OMDs showing the worst performance, followed by FMDs, who performed better than OMDs but worse than HCs. Conversely, stride time variability was significantly higher in FMDs than in both HCs and OMDs (p < 0.001; Table 4).

**TABLE 4:** Results of the statistical analysis for the spatio temporal parameters across the three experimental groups (HCs, FMDs, OMDs). Data are expressed as mean ± standard deviation. The level of statistical significance was set at p < 0.05; *post hoc* comparisons were corrected using the Bonferroni method.

Variables	Group	ST	mDT	cDT	vDT	Group effect p-value	Task effect p-value	T x G
Stride length	1.HC	1.27 ± 0.13	1.25 ± 0.14	1.20 ± 0.15	1.31 ± 0.13	**<0.001(1-2; 1-3);** ηp²**=** 0.101	**<0.001** (ST-mDT*; ST-cDT*; mDT-cDT*; mDT-vDT*; cDT-vDT*)	0.022*
	2.FMD	1.17 ± 0.27	1.11 ± 0.28	1.07 ± 0.25	1.15 ± 0.28			
	3.OMD	1.10 ± 0.19	1.08 ± 0.18	1.04 ± 0.18	1.13 ± 0.18			
Gait cycle duration	1.HC	1.14 ± 0.08	1.17 ± 0.10	1.23 ± 0.13	1.11 ± 0.13	**<0.001(1-2; 1-3);** ηp² **=** 0.131	**<0.001** (ST-mDT *; ST-cDT *; mDT-cDT *; mDT-vDT *; cDT-vDT *); **0.002** (ST-vDT) *	0.128
	2.FMD	1.20 ± 0.13	1.27 ± 0.16	1.33 ± 0.20	1.20 ± 0.13			
	3.OMD	1.21 ± 0.12	1.25 ± 0.12	1.33 ± 0.18	1.19 ± 0.11			
Speed	1.HC	1.11 ± 0.15	1.09 ± 0.15	0.98 ± 0.17	1.17 ± 0.14	**<0.001(1-2; 1-3);** ηp²**=** 0.174	**<0.001** (ST-mDT *; ST-cDT *; mDT-cDT*; mDT-vDT *; cDT-vDT *); **0.009** (ST-vDT)^A,C^	0.022*
	2.FMD	0.96 ± 0.30	0.89 ± 0.30	0.81 ± 0.27	0.96 ± 0.30			
	3.OMD	0.92 ± 0.19	0.87 ± 0.17	0.78 ± 0.20	0.94 ± 0.20			
Stride time variability	1.HC	3.00 ± 1.32	2.76 ± 1.26	3.16 ± 1.42	2.51 ± 1.14	**<0.001(1-2; 2-3);** ηp²**=** 0.137	**<0.001** (ST-cDT ^B,C^; mDT-cDT *; cDT-vDT*); **0.002** (ST-cDT ^A,C^; mDT-vDT ^A,B^)	**<0.001**
	2.FMD	3.68 ± 1.79	4.15 ± 2.14	4.20 ± 3.00	3.75 ± 2.17			
	3.OMD	3.20 ± 1.34	2.86 ± 1.39	4.00 ± 2.24	2.71 ± 1.13			

ST: Single task; mDT: motor dual-task; cDT: Cognitive dual-task; T: Task, G; Group; HC: Healthy control; FMD: Function motor disorders; OMD: Organic motor disorders. Bold p values indicate statistical significance (p < 0.05). Superscripts applied to the p values for the task effect indicate in which groups the variation between tasks was significant: * indicates a significant difference observed in all groups; a indicates a significant difference only in the HC group; b indicates a significant difference only in the FMD group; c indicates a significant difference only in the OMD group. ηp^2^ indicates partial eta squared, reported as a measure of effect size for the fixed effects of the model. According to Cohen’s conventions, ηp^2^ values of 0.01, 0.06, and 0.14 correspond approximately to small, medium, and large effects, respectively

A significant main effect of task was also observed, with differences across experimental conditions in all populations, as confirmed by pairwise comparisons (Table 4). In particular, stride time variability clearly distinguished FMDs from both HCs and OMDs (p < 0.001; Table 4), with FMD patients exhibiting a markedly irregular gait pattern characterized by variability levels significantly higher than those observed in both comparison groups (*post hoc*: p < 0.001; Table 4). Additionally, a significant group × task interaction was found for stride length, gait speed, and stride time variability (p = 0.022, p = 0.022, and p < 0.001, respectively; Table 4), indicating that performance under dual-task conditions differed across groups.

DTE analysis showed that dual-task conditions influenced gait parameters across all groups (see Table 5), with a general reduction in stride length during motor and cognitive dual tasks, although this effect was not statistically significant. In the visual dual-task, HCs and OMDs showed increased DTE for stride length, whereas FMDs exhibited negative DTE values for step length. A significant task effect was observed (p < 0.001), with no significant group effect or group × task interaction (Table 5).

**TABLE 5:** Results of the statistical analysis for the DTE on gait parameters across the three experimental groups (HCs, FMDs, OMDs). Data are expressed as mean ± standard deviation. The level of statistical significance was set at p < 0.05; *post hoc* comparisons were corrected using the Bonferroni method.

Variables	Group	mDT	cDT	vDT	Group effect p-value	Task effect p-value	T x G
DTE stride length	1.HC 2.FMD 3.OMD	−2.04 (0.68) −3.79 (1.59) −2.07 (0.90)	−5.55 (0.72) −7.09 (1.60) −5.34 (1.48)	3.32 (0.74)-0.50 (1.63) 2.27 (1.11)	0,177	**<0.001** (mDT-cDT*; cDT-vDT*; mDT-vDT*)	0.229
DTE gait cycle duration	1.HC 2.FMD 3.OMD	−2.51 (0.52) 5.59 (0.89) 2.78 (0.56)	8.28 (0.83) 11.47 (1.38) 9.76 (1.03)	-2.32 (0.88) -0.12 (0.87) -2.41 (0.61)	**0.004 (1-2);** ηp²**=** 0.026	**<0.001** (mDT-cDT*; cDT-vDT*; mDT-vDT*)	0.789
DTE speed	1.HC 2.FMD 3.OMD	−1.86 (0.89) −7.07 (1.62) −3.36 (1.21)	−11.53 (1.06) −13.93 (1.76) −14.78 (1.44)	5.90 (0.98) 1.65 (2.02) 3.65 (1.52)	0,064	**<0.001** (mDT-cDT*; cDT-vDT*; mDT-vDT*)	0.365
DTE stride time variability	1.HC 2.FMD 3.OMD	6.96 (6.68) 18.62 (6.81) -6,23 (2.76%)	22.82 (5.98) 20.55 (11.19) 25.20 (11.77)	0.05 (5.04) 1.63 (3,41) -3.57 (5.76)	**<0,001 (1-2; 2-3; 1-3);** ηp²**=** 0.016	0.752	0.235

DTE: dual task effect; motor dual-task; cDT: cognitive dual-task; vDT:visual Dual-Task; T: task, G; group; HC: healthy control; FMD: function motor disorders; OMD: Organic Motor Disorders. Superscripts applied to *post hoc* comparisons indicate in which groups the variation between tasks was significant: * indicates a significant difference observed in all groups; ^A^ indicates a significant difference only in the HC, group; ^B^ indicates a significant difference only in the FMD, group; ^C^ indicates a significant difference only in the OMD, group. ηp^2^ indicates partial eta squared, reported as a measure of effect size for the fixed effects of the model. According to Cohen’s conventions, ηp^2^ values of 0.01, 0.06, and 0.14 correspond approximately to small, medium, and large effects, respectively. Statistical Significance in bold

For step duration, DTE increased in all groups under dual-task conditions, with a significant difference between FMDs and HCs (p = 0.004; Table 5). A significant task effect was also observed, with no interaction effect. For gait speed, DTE decreased during motor and cognitive dual tasks and increased during the visual dual-task; no significant group effect was found (p = 0.064), while the task effect remained significant (p < 0.001; Table 5).

In contrast, DTE for gait variability increased in all dual-task conditions in FMDs, whereas OMDs showed mixed responses across tasks. A significant group effect was present (p < 0.001), with no significant task effect or interaction (Table 5).

#### 3.2.4 Exploratory subgroup analyses

Exploratory subgroup analyses did not identify statistically significant differences in gait parameters among the main clinical phenotypes within either the FMDs or OMDs cohorts (all *p* > 0.05). These findings are reported in the Supplementary Tables S7, S8.

#### 3.2.5 Sensitivity analyses

Sensitivity analyses were performed to evaluate whether the primary between-group findings for gait performance remained robust when considering more clinically homogeneous and phenotype-matched subgroups. Repeating the primary analyses separately in patients with essential tremor and dystonia yielded a pattern of results that was consistent with the primary analyses, with comparable magnitude and direction of the observed between-group differences (Supplementary Tables S9, S10).

Similarly, the phenotype-matched analysis including HCs, OMD patients with tremor, and FMD patients presenting tremor without concomitant dystonia yielded findings consistent with the primary analyses. Significant between-group differences in gait performance were maintained across the evaluated conditions, with medium-to-large effect sizes observed for several gait parameters (Supplementary Table S11).

### 3.3 Psychometric assessment

Psychometric assessment showed significant group differences for anxiety and depressive symptoms (Table 6). In particular, patients with FMDs reported higher BAI and BDI scores than both HCs and OMDs. Post hoc analyses confirmed significant differences between FMDs and HCs and between FMDs and OMDs (p < 0.001; Table 6). By contrast, TAS-20 scores, an index of alexithymia, were significantly different between healthy and clinical groups, with higher values in FMDs (p < 0.001; *post hoc* comparisons: HCs vs. FMDs and HCs vs. OMDs; Table 6), although no significant difference was found between the FMD and OMD groups. In addition, based on MFI scores, individuals with FMDs showed significantly higher levels of general fatigue compared with the other groups (p < 0.001; *post hoc* comparisons: HCs vs. FMDs and FMDs vs. OMDs; Table 6). Finally, SF-12 scores were significantly lower in both clinical groups compared with HCs, with additional differences between the two patient groups. For the physical component, FMDs showed lower scores than both HCs and OMDs (p < 0.001; *post hoc* comparisons: HCs vs. FMDs and FMDs vs. OMDs; Table 6). Similarly, for the mental component, FMD patients reported the lowest scores, clearly distinguishing them from both HCs and OMDs, with OMD patients showing mental health values closer to normative levels (p < 0.001; pairwise comparisons: HCs vs. FMDs and FMDs vs. OMDs; see Table 6).

**TABLE 6:** Results of the statistical analysis for the psychometric measurements across the three experimental groups (HC, FMD, OMD). Data are expressed as mean ± standard deviation. Effect sizes (Cohen’s d) are reported for statistically significant group pairwise comparisons. The level of statistical significance was set at p < 0.05; *post hoc* comparisons were corrected using the Bonferroni method.

Variables	1.HC (n = 153)	2.FMD (n = 149)	3.OMD (n = 99)	p-value	Cohen’s d
TAS-20 total score	39.52 ± 10.11	48.73 ± 14.67	47.98 ± 13.42	**<0.001 (1-3; 1-2)**	d_1-2_ = −0.72; d_1-3_ =−0.65
BAI total score	6.90 ± 5.80	20.13 ± 11.34	9.15 ± 6.96	**<0.001 (1-2; 2-3)**	d_1-2_ = −1.44; d_2-3_ = 1.13
BDI total score	5.37 ± 4.47	13.05 ± 8.90	6.42 ± 4.71	**<0.001 (1-2; 2-3)**	d_1-2_ = −1.13; d_2-3_ = 0.93
BDI somatic score	3.84 ± 3.37	8.54 ± 5.57	4.35 ± 3.20	**<0.001 (1-2; 2-3)**	d_1-2_ = −1.04; d_2-3_ = 0.87
BDI cognitive score	1.52 ± 1.75	4.22 ± 3.97	1.98 ± 1.97	**<0.001 (1-2; 2-3)**	d_1-2_ = −0.87; d_2-3_ = 0.66
SF-12 physical health score	53.99 ± 5.52	33.40 ± 10.27	43.44 ± 9.91	**<0.001 (1-2; 1-3; 2-3)**	d_1-2_ = 2.56; d_1-3_ = 1.39; d_2-3_ = −1.02
SF-12 mental health score	48.06 ± 8.84	41.42 ± 11.60	47.55 ± 9.60	**<0.001 (1-2; 2-3)**	d_1-2_ = 0.64; d_2-3_ = −0.55
MFI general fatigue	10.17 ± 3.86	15.11 ± 3.74	11.68 ± 4.16	**0.036 (1-3); <0.001 (1-2; 2-3)**	d_1-2_ = −1.29; d_1-3_ = −0.37; d_2-3_ = 0.85
MFI physical fatigue	8.89 ± 3.99	14.36 ± 3.99	11.40 ± 4.53	**<0.001 (1-3; 1-2; 2-3)**	d_1-2_ = -1.34; d_1-3_ = -0.57; d_2-3_ = 0.67
MFI reduced activity	8.13 ± 3.87	12.72 ± 4.75	10.53 ± 4.29	**<0.001 (1-3; 1-2); 0.003 (2-3)**	d_1-2_ = -1.06; d_1-3_ = -0.55; d_2-3_ = 0.47
MFI reduced Motivation	7.19 ± 2.85	9.49 ± 3.85	8.50 ± 3.17	**0.004 (1-3); <0.001 (1-2)**	d_1-2_ = −0.68; d_1-3_ =−0.45
MFI mental fatigue	9.39 ± 4.04	12.44 ± 4.48	9.43 ± 4.17	**<0.001 (1-2; 2-3)**	d_1-2_ = -0.69; d_2-3_ =0.67
MFI total score	43.88 ± 15.29	64.05 ± 16.45	51.88 ± 17.10	**0.003 (1-3); <0.001 (1-2; 2-3)**	d_1-2_ = −1.26; d_1-3_ = -0.46; d_2-3_ = 0.73
BAQ total score	79.80 ± 17.04	74.01 ± 18.68	76.59 ± 21.94	**0.032 (1-2)**	d_1-2_ = 0.32

S-FMDRS simplified functional movement disorders rating scale, MFI multidimensional fatigue inventory scale, SF-12 12-item short-form health survey, BAI beck anxiety inventory, BDI beck depression inventory, TAS-20 Toronto Alexithymia scale, BAQ body awareness questionnaire. According to Cohen’s conventions, d values of 0.2, 0.5, and 0.8 indicate small, medium, and large effects, respectively. Statistical Significance in bold.

Given the significant differences observed in psychometric measures across groups, we examined whether these variables were associated with motor performance. To this end, correlation analyses were performed between psychometric measures and both postural control and gait analysis variables.

#### 3.3.1 Psychometric correlates of postural control

Exploratory analyses revealed nominal associations between psychometric measures and postural control variables across groups, as illustrated in Figure 4. In the FMD group (Figure 4a), nominally significant positive correlations were observed between anxiety and depression levels (BAI and BDI) and all stabilometric parameters, with moderate effect sizes (ρ > 0.400; uncorrected p < 0.05 across conditions). In addition, somatic BDI scores showed nominal positive associations with sway area and perimeter across all conditions (uncorrected p < 0.05). Conversely, physical quality of life (SF-12 physical component) showed nominal negative correlations with all posturographic parameters (uncorrected p < 0.05), with stronger associations (ρ < −0.500) observed during dual-task conditions. General and physical fatigue showed exploratory associations with increased sway area in most conditions (EO and EC), particularly during the motor dual-task with EC (mDT-EC; ρ = 0.525, p < 0.01). Mental fatigue was also nominally associated with several posturographic parameters (uncorrected p < 0.05).

**FIGURE 4:**
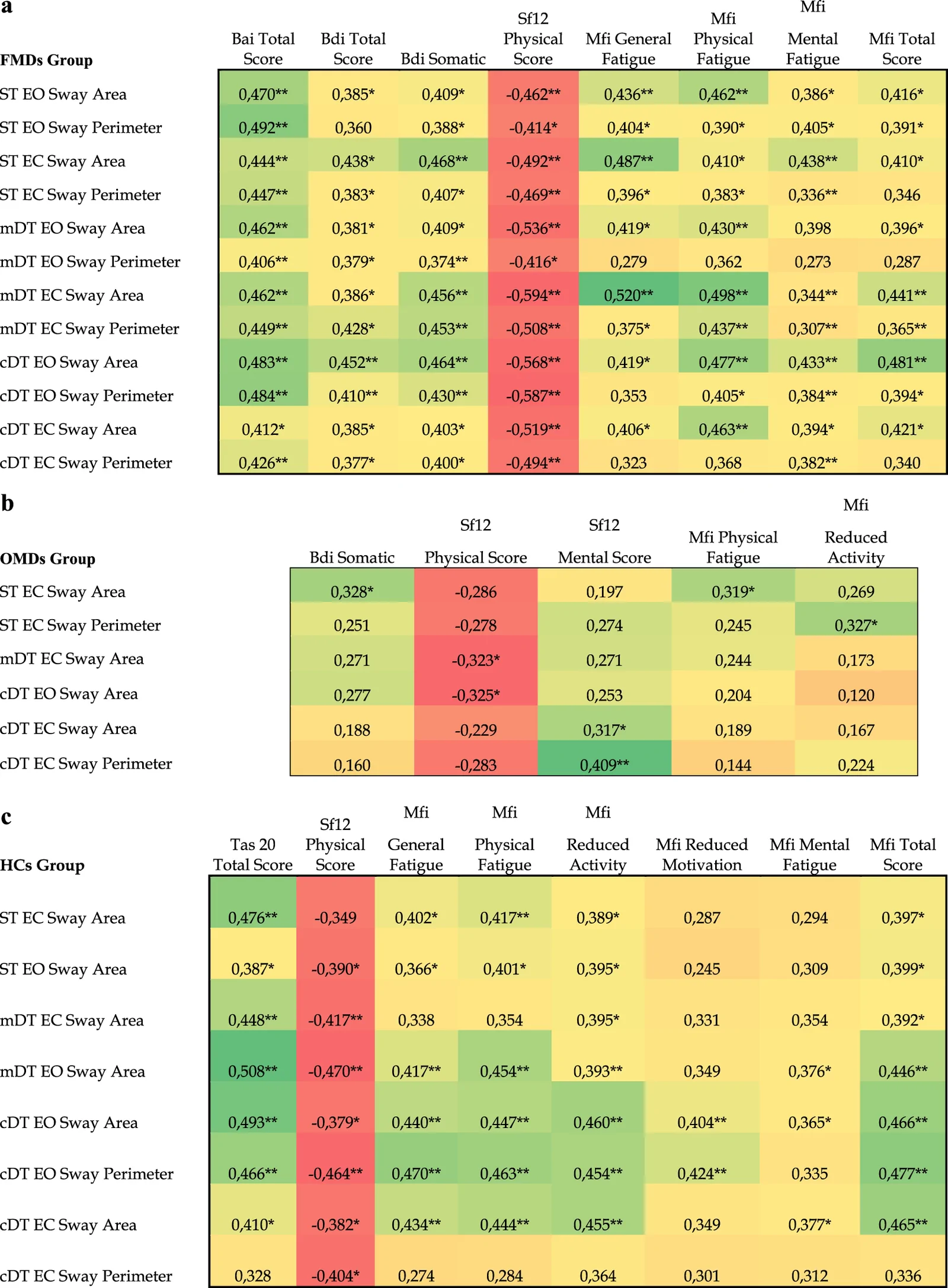
**(a-c)** Heatmaps of correlation coefficients between psychometric measures and postural control parameters in the FMDs, OMDs, and HCs groups. Specifically, **(a)** refers to the FMDs group, **(b)** to the OMDs group, and **(c)** to the HCs group. Color intensity indicates the strength and direction of the correlations: green denotes positive correlations, whereas red denotes negative correlations.

Lower and more selective correlations were observed in the OMD group (Figure 4b). Specifically, somatic BDI scores showed a nominal positive correlation with sway area in the single-task EC condition (ST-EC; ρ = 0.328, uncorrected p < 0.05). Similarly, physical fatigue (MFI) was positively correlated with sway area in the ST-EC condition (ρ = 0.319, p < 0.05), while a comparable correlation was observed for sway perimeter in the same condition (ρ = 0.327, p < 0.05). In contrast, the SF-12 physical component score showed nominal negative correlations with postural sway during dual-task conditions, suggesting a possible relationship between lower physical quality of life scores and increased sway area during the motor dual-task with EC (mDT-EC; ρ = −0.323, p < 0.05) and the cognitive dual-task with EO (cDT-EO; ρ = −0.325, p < 0.05). Conversely, the SF-12 mental component score showed nominal positive associations with instability during more demanding conditions, with correlations observed for both sway area (ρ = 0.317, p < 0.05) and perimeter (ρ = 0.409, p < 0.01) during the cognitive dual-task with EC (cDT-EC).

In the HCs group (Figure 4c), a nominal positive correlation was observed between total TAS-20 scores and several postural stability parameters, with the strongest associations observed for sway area in both single- and dual-task conditions (uncorrected p < 0.05). These correlations were evident under both EO and EC conditions (ρ > 0.400; p < 0.05). In contrast, the SF-12 physical component score showed nominal negative correlations across nearly all conditions, indicating that lower physical quality of life was consistently associated with greater postural sway, with the strongest correlation observed during the motor dual-task with EC (mDT-EC; ρ = −0.470, p < 0.01). Furthermore, total MFI scores and its subcomponents showed multiple significant associations with postural control parameters. In particular, general and physical fatigue were nominally correlated with increased sway during the cognitive dual-task in both EO (ρ = 0.440 and ρ = 0.447, p < 0.01) and EC conditions (ρ = 0.434 and ρ = 0.444, p < 0.01), as well as during the motor dual-task with EC (ρ = 0.417 and ρ = 0.454, p < 0.01). Reduced activity was also significantly correlated with most stabilometric parameters (p < 0.05 or p < 0.01). Overall, the observed associations between MFI total score and postural instability suggest a potential relationship between fatigue perception and postural control during cognitive dual-task (ρ = 0.477, p < 0.01).

However, after correction for multiple comparisons, none of the observed correlations remained statistically significant. Therefore, these findings should be interpreted as exploratory.

#### 3.3.2 Psychometric correlates of gait analysis

Exploratory analyses revealed nominal associations between psychometric measures and gait variables across groups (Figure 5). In the FMD group (Figure 5a), nominal associations were observed between gait parameters and measures of physical quality of life (SF-12 Physical) and fatigue (MFI). In particular, higher SF-12 Physical scores were nominally associated with higher gait performance-related measures across all conditions (single, motor, cognitive, and visual dual-tasks; p < 0.05). Specifically, better physical health was associated with longer stride lengths (up to ρ = 0.580, p < 0.01) and higher gait speed (up to ρ = 0.588, p < 0.01). Conversely, physical fatigue and reduced activity showed nominal negative associations with shorter stride length and lower gait speed, with stronger effects during the visual dual-task (stride length: ρ = −0.401 and −0.406, respectively; p < 0.01). Mental fatigue also showed nominal associations with gait parameters during more complex conditions, correlating with reduced stride length in both the cognitive dual-task (ρ = −0.351, p < 0.01) and visual dual-task (ρ = −0.274, p < 0.01). Finally, increased stride time variability was significantly associated with lower physical quality of life, particularly during the visual dual-task (ρ = −0.303, p < 0.01).

**FIGURE 5:**
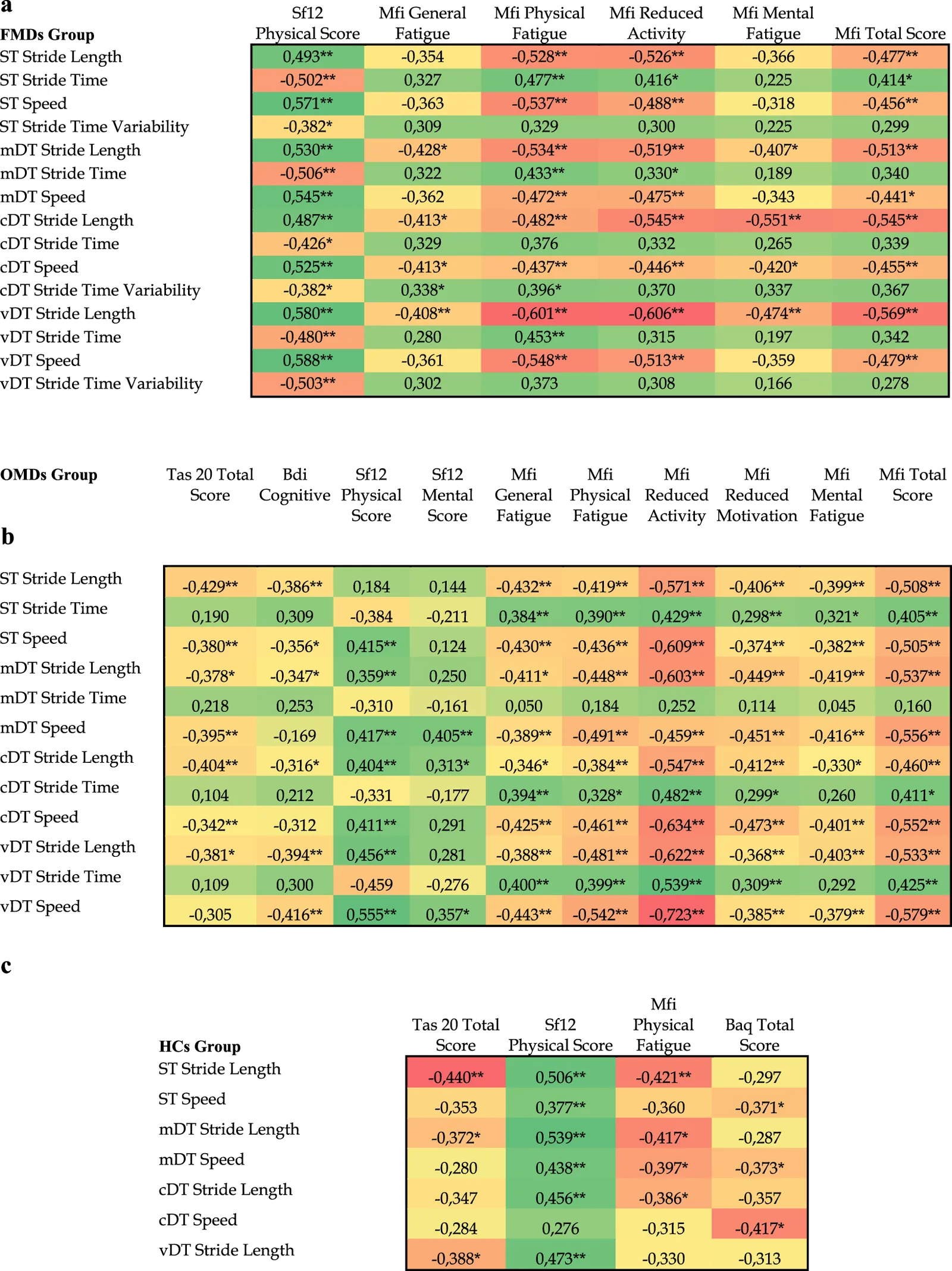
**(a-c)** Heatmaps of correlation coefficients between psychometric measures and gait parameters in the FMDs, OMDs, and HCs groups. Specifically, **(a)** refers to the FMDs group, **(b)** to the OMDs group, and **(c)** to the HCs group. Color intensity indicates the strength and direction of the correlations: green denotes positive correlations, whereas red denotes negative correlations.

In the OMD group (Figure 5b), higher TAS-20 scores and cognitive BDI scores were nominally associated with reduced stride length and slower gait speed across all conditions (ρ > −0.300, p < 0.05). Conversely, both physical and mental health scores were positively associated with increased gait speed and stride length (p < 0.05). Furthermore, reduced activity (MFI) was negatively correlated with gait speed across all phases, reaching a peak of ρ = −0.723 (p < 0.01) during the visual dual-task. Physical fatigue and the MFI total score also showed significant associations with increased stride time and reduced speed and stride length (p < 0.01).

In the HCs group (Figure 5c), the SF-12 Physical component showed the most consistent nominal positive correlation pattern with gait efficiency across nearly all conditions (p < 0.01). Higher physical health was nominally associated with longer stride length in the single-task (ρ = 0.506, p < 0.01), motor dual-task (ρ = 0.539, p < 0.01), cognitive dual-task (ρ = 0.456, p < 0.01), and visual dual-task (ρ = 0.273, p < 0.01) conditions. In addition, higher TAS-20 scores were associated with reduced stride length, particularly during the single-task (ρ = −0.440, p < 0.01) and visual dual-task (ρ = −0.388, p < 0.05). Moreover, increased physical fatigue showed nominal negative associations with shorter stride length across single-task (ρ = −0.121, p < 0.01), motor dual-task (ρ = −0.417, p < 0.01), and cognitive dual-task (ρ = −0.386, p < 0.05) conditions, and also negatively affected gait speed during the motor dual-task (ρ = −0.397, p < 0.05). Although showing fewer associations, BAQ scores were significantly correlated with gait speed during the cognitive dual-task (ρ = −0.417, p < 0.05).

However, after correction for multiple comparisons, none of the observed associations remained statistically significant. Therefore, these findings should be interpreted as exploratory.

## 4 Discussion

In this study, we comprehensively characterize the gait and postural control patterns of patients with FMDs compared to OMDs and HC using a standardized experimental protocol that combines dual-task conditions and sensory manipulation. The inclusion of a heterogeneous group of OMDs was intended to better match the clinical and phenotypic variability observed in FMDs, thereby supporting a more appropriate comparison for diagnostic differentiation. Beyond the objective description of gait and postural control performance, our findings, in a large multicenter cohort, highlight a distinct motor and psychometric pattern in FMDs that differentiates this group not only from HC but, more importantly, from OMDs. This latter comparison is still poorly explored in the literature.

The differences observed in our findings in the distribution by age and sex between the FMDs and OMDs groups reflect the epidemiological characteristics of FMDs, which predominantly affect women with a typical age of onset between 40 and 50 years (Baizabal-Carvallo and Jankovic, 2020; Espay et al., 2018). Beyond demographic characteristics, FMDs showed a more pronounced impairment in postural control compared to both HCs and OMDs, as reflected by increased sway area and perimeter across all experimental conditions, suggesting a general alteration in postural stability.

Across groups, dual-task conditions were associated with the expected attentional cost, consistent with the cognitive demands of postural control. However, this pattern was less consistent in FMDs, particularly under EC conditions, where patients showed relatively smaller deteriorations and, in some cases, reduced sway area during dual-task performance. Notably, cognitive performance in FMDs remained stable across EO and EC conditions, suggesting that the improvement in postural control under EC conditions was not accompanied by additional cognitive costs (Supplementary Table S12). In this context, dual-task conditions may modulate postural control by altering attentional resource allocation and reducing excessive self-monitoring, in line with current models of high-level sensorimotor control in functional gait and balance disorders (Sandri et al., 2025). The most relevant finding concerns the differential response to dual-task conditions in FMDs, particularly when compared to OMDs. Indeed, DTE emerged as a key discriminative feature, with FMDs showing a paradoxical or reduced DTE, in contrast to the deterioration observed in both OMDs and HCs. Consistently, DTE results indicate group-specific differences in dual-task modulation, with FMDs showing a reduction of the typical dual-task cost, particularly during cognitive loading. These findings, together with significant Group × Task interactions, may suggest distinct postural control strategies across groups. In particular, the elevated Romberg index may indicate a greater dependence on visual input and altered sensory integration processes in FMDs, supporting the hypothesis of differential interaction between attentional load and motor control rather than a simple reflection of overall motor impairment severity. Sway area under EC conditions further may emerge as a potentially informative marker for group differentiation.

In regard to gait performance, FMDs showed less severe deficits in spatiotemporal gait parameters compared to OMDs, but greater impairments relative to HCs, with the exception of stride time variability, which emerged as the most sensitive marker, clearly distinguishing FMDs from both groups. These findings were also confirmed in a subgroup analysis including FMD and HC participants matched for height, indicating that the observed differences were not attributable to anthropometric factors (Supplementary Table S13). Furthermore, when the comparison was repeated in a subgroup additionally matched for gait speed, most of the significant differences remained evident, supporting the robustness of the findings beyond the influence of walking velocity (Supplementary Table S14). Across all participants, dual-task conditions were associated with the expected attentional cost, reflected by reduced gait speed, shorter stride length, and increased stride duration, particularly during cognitive dual-tasking. However, a different pattern emerged in terms of DTE. FMDs showed a maladaptive or atypical response, characterized by increased gait variability and, in some conditions, negative DTE values. In contrast, HCs and OMDs generally exhibited the expected dual-task cost, suggesting a more typical motor–cognitive interaction strategy. Consistently, significant Group × Task interactions indicate that adaptation strategies differed across groups, with visual dual-task conditions appearing to facilitate gait performance in HCs and OMDs but not in FMDs, suggesting potential differences in sensory integration mechanisms.

Within this context, exploratory correlation analyses identified nominal associations between motor performance and psychometric measures in FMDs, in line with recent evidence linking neurophysiological markers with affective and interoceptive domains in this population (Vinciguerra et al., 2026). In FMDs, postural control showed exploratory associations mainly with anxiety, depression, and fatigue, while gait performance was primarily related to fatigue and physical quality of life. No relevant association was observed between alexithymia and motor performance in this group. Conversely, exploratory correlations suggested possible relationships between alexithymia and gait performance in OMDs and HCs, as well as with postural stability in controls. These results are consistent with the possibility that motor performance in FMDs may be related to a different constellation of clinical factors compared to organic conditions and HCs.

To our knowledge, only a few studies have examined postural and gait deficits in patients with FMDs. Gandolfi et al. demonstrated that performing a cognitive or motor dual-task alongside a postural or gait task can improve stability and performance in patients with FMDs. In contrast, HCs show minimal changes (Gandolfi et al., 2023; 2021a). This pattern has been interpreted as potentially reflecting a reduction in excessive internal attentional focus during dual-task conditions, which may be associated with the observed improvements in postural control and gait.

Psychometric assessment further highlighted the greater non-motor burden characterizing FMDs, including higher levels of anxiety, depression, and fatigue, together with poorer quality of life. Within this context, exploratory correlation analyses revealed potential associations between motor performance and psychometric measures in FMDs. In particular, higher anxiety and depression scores were associated with greater postural instability, as reflected by increased sway area and perimeter. These observations are consistent with previous evidence linking affective symptoms to impaired motor performance and postural control (Elkjær et al., 2022; Rossato et al., 2026). Although participants with diagnosed major depressive or anxiety disorders were excluded, subclinical affective symptoms may still contribute to motor dysfunction and represent an important component of the clinical profile of FMDs.

Fatigue emerged as a relevant factor in FMDs, showing exploratory associations with both postural and gait performance. Higher levels of overall, physical, and mental fatigue were associated with greater postural instability, whereas better physical quality of life was associated with trends toward more stable postural control. Similarly, increased fatigue was related to poorer gait performance, characterized by lower gait speed, shorter step length, and greater gait variability, particularly during visually demanding tasks.

We also explored, for the first time, the relationship between alexithymia and motor performance. Alexithymia, defined as difficulty identifying and describing emotions (Ostuzzi et al., 2025), showed exploratory associations with poorer gait performance in both HCs and OMDs and with reduced postural stability in HCs. In contrast, no clear association was observed between alexithymia and motor performance in FMDs. These preliminary findings suggest that emotional processing traits may represent a potential factor influencing motor performance in non-functional populations, whereas motor dysfunction in FMDs may be more closely related to other clinical factors (Ostuzzi et al., 2025). However, these observations require confirmation in future studies.

The study limitations include the cross-sectional design, which limits causal inference, and the potential influence of residual confounding factors despite their inclusion as covariates. In particular, group differences in demographic characteristics, such as age, gender and education level, may have partially influenced the observed outcomes, although these variables were accounted for in the statistical analyses. In addition, some participants were unable to complete the gait assessments, due to motor impairment preventing them from safely completing the walking protocol.

Additionally, although the inclusion of different OMDs phenotypes was intended to reflect the clinical heterogeneity of FMDs in the differential diagnosis, this approach may have introduced variability related to disorder-specific effects. Therefore, to evaluate the robustness of the primary findings, we performed both exploratory subgroups and sensitivity analyses (Supplementary Tables S2–S11). The exploratory analyses did not identified significant differences among the main clinical phenotypes within the FMDs and OMDs cohorts, whereas the sensitivity analyses yielded findings consistent with the primary analyses when more homogeneous OMDs subgroups and a phenotype-matched tremor subgroup were considered. Nevertheless, these analyses remain exploratory and were limited by the relatively small size of some clinical subgroups, particularly within the FMDs cohort. Therefore, larger studies including more balanced phenotype-specific populations will be necessary to confirm the robustness and generalizability of these findings and to better characterize phenotype- and disorder-specific mechanisms underlying postural control and gait impairment in FMDs and OMDs.

Furthermore, the exploratory correlation analyses between psychometric characteristics and instrumental motor outcomes should be interpreted cautiously, as they were primarily intended to identify potential relationships and generate new hypotheses regarding the interplay between psychological factors and motor manifestations in FMDs and related disorders. Future longitudinal studies will be required to validate these preliminary observations and to clarify the mechanisms underlying these potential associations. Because the study was conducted in two tertiary referral centres, caution is warranted when generalizing these findings to other clinical settings.

To our knowledge, this is the first study to objectively evaluate gait and posture control in a large cohort of FMDs patients, compared with OMDs and HCs, using standardized quantitative instrumental analysis of gait and posture. Our findings indicate that specific quantitative gait and postural parameters such as DTE, sway area under eyes-closed conditions, and stride time variability, may represent promising candidate markers for the differential diagnosis of FMDs against organic movement disorders.

## Data Availability

The datasets presented in this article are not readily available due to privacy reasons. Requests to access the datasets should be directed to the corresponding authors.
