# Supplementary material for: Quantitative analysis of posture and gait in the differential diagnosis of functional and organic motor disorders: a multicenter cross-sectional study
Source: Front Bioeng Biotechnol. 2026 Oct 5;14:1872332. doi: 10.3389/fbioe.2026.1872332 (PMC13677921; doi:10.3389/fbioe.2026.1872332)
Supplement: Supplementary file 1 [file Supplementaryfile1.pdf]

SUPPLEMENTARY MATERIALS

Quantitative Analysis of Posture and Gait in the Differential Diagnosis of Functional and Organic Motor Disorders: A Multicenter cross-sectional Study

***Michela Russo[1] michela.russo2@unina.it**
***Roberto Erro[2,3,4] roberto.erro@gmail.com**
Carlo Ricciardi [5] carlo.ricciardi@unina.it
Marialuisa Gandolfi[6,7] marialuisa.gandolfi@univr.it
Angela Sandri[7] angela.sandri01@univr.it
Marianna Amboni[3] mamboni@unisa.it
Elisabetta Sarasso[8,9,10] sarasso.elisabetta@hsr.it
Andrea Gardoni[8,9] Gardoni.Andrea@hsr.it
Silvia Basaia[8,9] basaia.silvia@hsr.it
Sofia Cuoco[3] dott.sofiacuoco@gmail.com
Immacolata Carotenuto[3] carotenutoimma@gmail.com
Claudia Vinciguerra[2] claudiavinci@hotmail.it
Annibale Botto[11] annibale.botto@gmail.com
Daniele Romano[11] daniele.romano@sangiovannieruggi.it
Ilaria Antonella Di Vico[7] ilariadivico89@gmail.com
Mirta Fiorio[6] mirta.fiorio@univr.it
Serena Camozzi[7] serena.camozzi@univr.it
Giulia Pedrotti[6] giulia.pedrotti_02@univr.it
Anna Fratucello[7] anna.fratucello@aovr.veneto.it
Giancarlo Mansueto[12,13] giancarlo.mansueto@univr.it
Francesca Benedetta Pizzini[16] francescabenedetta.pizzini@univr.it
Marco Barillari[12] marco.barillari@univr.it
Matteo Francesco Lauriola[7] matteofrancesco.lauriola@aovr.veneto.it
Mariachiara Tozzi[7] mariachiara.tozzi@aovr.veneto.it
Francesca Rusciano[7] francesca.rusciano@aovr.veneto.it
Christian Geroin[14] christian.geroin@univr.it
Melania Fasoli[6] melania.fasoli@univr.it
Angela Marotta[6] angela.marotta@univr.it
Francesca Salaorni[6] francesca.salaorni_02@univr.it
Giovanna Maddalena Squintani[7] giovannamaddalena.squintani@aovr.veneto.it
Sara Mariotto[7] sara.mariotto@aovr.veneto.it
Stefano Tamburin[6] stefano.tamburin@univr.it
Fabio Paio[6] fabio.paio1710@gmail.com
Giuseppe De Biasi[3] gdebiasi@unisa.it
Giuseppe Piscosquito[2] g.piscosquito@sangiovannieruggi.it
Elisa Sibilla[8] sibilla.elisa@hsr.it
Elisa Canu [8,9] canu.elisa@hsr.it
Massimo Filippi[8,9,15,16,17,18] filippi.massimo@hsr.it
Federica Agosta[8,9,15,18] agosta.federica@hsr.it
Maria Romano[5] mariarom@unina.it
Paolo Barone[2,3,4] pbarone@unisa.it
Francesco Amato[5] framato@unina.it
***Michele Tinazzi[6,7] michele.tinazzi@univr.it**
***Maria Teresa Pellecchia[2,3,4] mpellecchia@unisa.it**
***equally contributed**

[1]Department of Chemical, Material and Industrial Production Engineering, University of Naples Federico II, Naples, Italy,
[2]Neurology Unit, University Hospital "San Giovanni di Dio e Ruggi d'Aragona", Salerno, Italy,
[3]Department of Medicine, Surgery and Dentistry, "Scuola Medica Salernitana", University of Salerno, Baronissi, Italy,
[4]IRCCS SYNLAB SDN, Naples, Italy
[5]Department of Electrical Engineering and Information Technologies, University of Naples Federico II, Naples, Italy,
[6]Department of Neurosciences, Biomedicine and Movement Sciences, University of Verona, Verona, Italy,

[7]Azienda Ospedaliera Universitaria Integrata Verona, Verona, Italy,
[8]Neuroimaging Research Unit, Division of Neuroscience, IRCCS San Raffaele Scientific Institute, Milan, Italy,
[9]Neurotech Hub, Vita-Salute San Raffaele University, Milan, Italy,
[10]Department of Neuroscience, Rehabilitation, Ophthalmology, Genetics and Maternal Child Health, University of Genoa, Genoa, Italy,
[11]UOC Neuroradiologia Diagnostica ed Interventistica Azienda Ospedaliera-Universitaria "San Giovanni di Dio e Ruggi d'Aragona", Salerno, Italy,
[12]Department of Diagnostic and Public Health, Section of Radiology, University of Verona, Verona, Italy,
[13]Department of Radiology, Ospedale G.B. Rossi AOUI Verona, Verona, Italy,
[14]Department of Surgery, Dentistry, Pediatrics, and Gynecology, University of Verona, Verona, Italy,
[15]Neurology Unit, IRCCS San Raffaele Scientific Institute, Milan, Italy,
[16]Neurorehabilitation Unit, IRCCS San Raffaele Scientific Institute, Milan, Italy
[17]Neurophysiology service, IRCCS San Raffaele Scientific Institute, Milan, Italy,
[18]Vita-Salute San Raffaele University, Milan, Italy.

***** Corresponding Author
michela.russo2@unina.it
Keywords: Functional Motor Disorders, Organic Motor Disorders, Postural Control, Gait Analysis, Quantitative Assessment

List of anonymous

- Functional Movement Disorders (FMDs)
- Organic Movement Disorders (OMDs)
- Heatly Control (HCs)

Table S1 Main interpretation: Clinical symptom distribution in the FMDs cohort.

Table S1. Clinical symptom distribution in the FMDs cohort. Values are reported as number of patients and percentage of the total FMD cohort. As patients could present with more than one functional motor symptom, percentages do not sum to 100%.

Symptom	FMD, n (%)
Weakness	94 (62.7)
Gait disorders	80 (53.3)
Tremor	77 (51.3)
Dystonia	50 (33.3)
Myoclonus	24 (16.0)
Other	66 (44.0)

Table S2 Main interpretation: Postural control performance was comparable in the OMDs group between essential tremor and dystonia subgroups. The analysis showed no significant between-group differences detected in any condition.

Table S2. Comparison between OMDs sub-groups for postural control parameters.

	Essential Tremor Mean ± SD (n=44)	Dystonia Mean ± SD (n =52)	p-value
	**Single Task**		
**EO sway area**	211,10 ± 117,00	207,29 ± 114,87	0,898
**EO sway perimeter**	336,01 ± 71,39	322,98 ± 86,03	0,463
**EC sway area**	448,10 ± 279,35	457,12 ± 252,91	0,603
**EC sway perimeter**	590,97 ± 248,22	576,87 ± 220,34	0,777
**Romberg index (%)**	223,57 ± 105,03	217,23 ± 100,72	0,552
	**Motor Dual Task**		
**EO sway area**	247,34 ± 139,94	260,40 ± 130,12	0,338
**EO sway perimeter**	415,93 ± 112,66	451,74 ± 151,89	0,963
**EC sway area**	451,91 ± 279,05	475,77 ± 271,37	0,553
**EC sway perimeter**	641,26 ± 206,76	689,05 ± 243,72	0,607
**Romberg index (%)**	196,20 ± 101,42	201,21 ± 95,07	0,849
	**Cognitive Dual Task**		
**EO sway area**	298,90 ± 145,99	322,14 ± 154,55	0,416
**EO sway perimeter**	455,19 ± 147,57	444,71 ± 135,17	0,937
**EC sway area**	439,47 ± 315,55	433,70 ± 286,52	0,404
**EC sway perimeter**	612,92 ± 223,26	562,67 ± 198,87	0,463
**Romberg index (%)**	155,99 ± 67,73	130,75 ± 55,79	0,254

Table S3 Main interpretation: Postural control performance was comparable among FMDs subgroups (isolated motor manifestations): No significant differences emerged in postural control across single-task and dual-task conditions.

Table S3. Comparison between FMDs sub-groups for postural control parameters

	Weaknesses Mean ± SD (n=7)	Tremor Mean ± SD (n=7)	Dystonia Mean ± SD (n=7)	Gait Impairment Mean ± SD (n=7)	p-value
			**Single Task**		
**EO sway area**	218,85 ± 113,85	345,71 ± 371,09	222,60 ± 118,83	404,27 ± 154,96	0,345
**EO sway perimeter**	268,10 ± 97,04	427,43 ± 405,44	290,93 ± 107,00	328,01 ± 83,10	0,616
**EC sway area**	1248,18 ± 687,25	701,34 ± 425,90	481,79 ± 423,77	1015,86 ± 413,63	0,148
**EC sway perimeter**	619,95 ± 342,69	537,36 ± 208,91	456,33 ± 206,85	722,78 ± 256,93	0,454
**Romberg index (%)**	343,35 ± 137,00	338,30 ± 188,08	208,34 ± 143,53	294,84 ± 173,51	0,236
			**Motor Dual Task**		
**EO sway area**	230,66 ± 150,99	337,15 ± 177,17	308,69 ± 320,30	234,05 ± 67,09	0,705
**EO sway perimeter**	421,56 ± 172,50	582,74 ± 140,60	415,90 ± 182,31	631,69 ± 470,64	0,329

**EC sway area**	1283,09 ± 1702,53	555,11 ± 337,57	485,56 ± 334,73	1288,97 ± 485,02	0,213
**EC sway perimeter**	707,35 ± 339,88	718,59 ± 138,26	516,84 ± 188,00	1030,73 ± 222,29	0,058
**Romberg index (%)**	200,44 ± 72,53	149,21 ± 42,29	204,97 ± 136,15	312,17 ± 113,31	0,117
			**Cognitive Dual Task**		
**EO sway area**	273,49 ± 165,28	483,90 ± 435,71	338,97 ± 207,44	302,90 ± 233,45	0,869
**EO sway perimeter**	317,84 ± 112,69	440,37 ± 158,04	359,35 ± 93,73	383,34 ± 278,01	0,386
**EC sway area**	414,86 ± 296,75	511,72 ± 444,07	644,55 ± 527,55	401,59 ± 153,26	0,914
**EC sway perimeter**	425,71 ± 166,10	534,63 ± 252,59	496,81 ± 137,15	448,55 ± 205,69	0,759
**Romberg index (%)**	158,74 ± 93,83	180,08 ± 101,97	148,90 ± 78,77	166,91 ± 66,95	0,929

Table S4 Main interpretation: Postural control performance was comparable among HCs, FMDs, and OMDs with tremor phenotypes: statistically significant differences emerged in postural control across single-task and dual-task conditions. Patients with presence of isolated or combined dystonic phenotypes have been included in the analysis.

Table S4. Comparison between HCs vs FMDs vs OMDs Tremor Sub-group for postural control parameters.

	**1. HC Mean ± SD (n = 153)**	**2. FMD Mean ± SD (n = 149)**	**3. OMD - Tremor Mean ± SD (n=44)**	**p-value post-hoc**	**Cohen's d**
			**Single Task**		
**EO sway area**	166.19 ± 84.61	447.13 ± 405.27	211,10 ± 117,00	**<0,001(1-2; 2-3);**	d_{1-2}= -0.97; d_{2-3} = 0.65
**EO sway perimeter**	257.59 ± 63.99	412.43 ± 235.99	336,01 ± 71,39	**<0,001(1-2;1-3)**	d_{1-2}= -0.90; d_{2-3} = -1.19
**EC sway area**	348.58 ± 226.25	1353.25 ± 1261.73	448,10 ± 279,35	**<0,001(1-2; 2-3)**	d_{1-2}= -1.12; d_{2-3} = 0.81
**EC sway perimeter**	444.98 ± 158.55	753.86 ± 449.04	590,97 ± 248,22	**<0,001(1-2;1-3)**	d_{1-2}= -0.92; d_{1-3} = -0.80
**Romberg index (%)**	205.36± 91.00	290.01± 166.39	223,57 ± 105,03	**<0,001(1-2; 2-3)**	d_{1-2}= -0.60; d_{2-3} = 0.43
			**Motor Dual task**		
**EO sway area**	211.45 ± 92.18	461.53 ± 393.40	247,34 ± 139,94	**<0,001(1-2; 2-3)**	d_{1-2}= -0.82; d_{2-3} = 0.68
**EO sway perimeter**	466.90 ± 165.81	686.04 ± 438.26	415,93 ± 112,66	**<0,001(1-2; 2-3)**	d_{1-2}= -0.59; d_{2-3} = 0.83
**EC sway area**	398.55 ± 193.70	1009.96 ± 927.04	451,91 ± 279,05	**<0,001(1-2; 2-3)**	d_{1-2}= -0.83; d_{2-3} = 0.78
**EC sway perimeter**	637.40 ± 259.72	964.82 ± 560.78	641,26 ± 206,76	**<0,001(1-2; 2-3)**	d_{1-2}= -0.55; d_{2-3} =0.71
**Romberg index (%)**	174.48 ± 81.59	199.34 ± 96.94	196,20 ± 101,42	0,186	-
			**Cognitive dual task**		
**EO sway area**	224.95 ± 157.96	498.26 ± 389.04	298,90 ± 145,99	**<0,001(1-2; 2-3)**	d_{1-2}= -0.89; d_{2-3} = 0.65
**EO sway perimeter**	326.31 ± 91.91	455.32 ± 198.25	455,19 ± 147,57	**<0,001(1-2;1-3)**	d_{1-2}= -0.82; d_{1-3} = -1.14
**EC sway area**	285.43 ± 159.22	776.46 ± 702.68	439,47 ± 315,55	**<0,001(1-2;1-3;2-3)**	d_{1-2}= -0.92; d_{1-3} = -0.51; d_{2-3} = 0.62
**EC sway perimeter**	443.93 ± 147.24	642.85 ± 344.99	612,92 ± 223,26	**<0,001(1-2;1-3)**	d_{1-2}= -0.70; d_{1-3} = -0.75
**Romberg index (%)**	142.65 ± 92.73	151.54 ± 80.66	155,99 ± 67,73	0,058	-

Table S5 Main interpretation: Postural control performance was comparable among HCs, FMDs, and OMDs with dystonia phenotypes: statistically significant differences emerged in postural control across single-task and dual-task conditions. Patients with presence of isolated or combinated diatonic phenotype have been included in the analysis.

Table S5. Comparison between HCs vs FMDs vs OMDs Dystonia Sub-group for postural control parameters.

	**1. HC Mean ± SD (n = 153)**	**2. FMD Mean ± SD (n = 149)**	**3.OMD - Dystonia Mean ± SD (n = 52)**	**p-value post-hoc**	**Cohen's d**
			**Single Task**		
**EO sway area**	166.19 ± 84.61	447.13 ± 405.27	207,29 ± 114,87	**<0,001(1-2; 2-3)**	d_{1-2}= -0.97; d_{2-3} = 0.66
**EO sway perimeter**	257.59 ± 63.99	412.43 ± 235.99	322,98 ± 86,03	**<0,001(1-2;1-3)**	d_{1-2}= -0.90; d_{2-3} = -0.99
**EC sway area**	348.58 ± 226.25	1353.25 ± 1261.73	457,12 ± 252,91	**<0,001(1-2; 2-3;1-3)**	d_{1-2}= -1.12; d_{2-3} = 0.84
**EC sway perimeter**	444.98 ± 158.55	753.86 ± 449.04	576,87 ± 220,34	**<0,001(1-2;1-3)**	d_{1-2}= -0.92; d_{1-3} = -0.67
**Romberg index (%)**	205.36± 91.00	290.01± 166.39	217,23 ± 100,72	**<0,001(1-2; 2-3)**	d_{1-2}= -0.63; d_{2-3} = 0.43
			**Motor Dual task**		
**EO sway area**	211.45 ± 92.18	461.53 ± 393.40	260,40 ± 130,12	**<0,001(1-2; 2-3)**	d_{1-2}= -0.83; d_{2-3} = 0.67
**EO sway perimeter**	466.90 ± 165.81	686.04 ± 438.26	451,74 ± 151,89	**<0,001(1-2; 2-3)**	d_{1-2}= -0.59; d_{2-3} = 0.55
**EC sway area**	398.55 ± 193.70	1009.96 ± 927.04	475,77 ± 271,37	**<0,001(1-2; 2-3)**	d_{1-2}= -0.83; d_{2-3} = 0.73
**EC sway perimeter**	637.40 ± 259.72	964.82 ± 560.78	689,05 ± 243,72	**<0,001(1-2; 2-3)**	d_{1-2}= -0.55; d_{2-3} =0.59
**Romberg index (%)**	174.48 ± 81.59	199.34 ± 96.94	201,21 ± 95,07	0,159	-
			**Cognitive dual task**		
**EO sway area**	224.95 ± 157.96	498.26 ± 389.04	322,14 ± 154,55	**<0,001(1-2; 2-3;1-3)**	d_{1-2}= -0.89; d_{2-3} = 0.54; d_{1-3} = -0.62
**EO sway perimeter**	326.31 ± 91.91	455.32 ± 198.25	444,71 ± 135,17	**<0,001(1-2;1-3)**	d_{1-2}= -0.82; d_{1-3} = -1.01

**EC sway area**	285.43 ± 159.22	776.46 ± 702.68	433,70 ± 286,52	**<0,001(1-2;1-3;2-3)**	d_{1-2}= -0.92; d_{1-3} = -0.49; d_{2-3} = 0.61
**EC sway perimeter**	443.93 ± 147.24	642.85 ± 344.99	562,67 ± 198,87	**<0,001(1-2;1-3)**	d_{1-2}= -0.70; d_{1-3} = -0.51
**Romberg index (%)**	142.65 ± 92.73	151.54 ± 80.66	130,75 ± 55,79	0,138	-

Table S6 Main interpretation: Postural control performance was compared among HCs, FMD patients presenting tremor symptoms (including patients with additional motor manifestations but without dystonia), and OMD patients with tremor manifestations.

Table S6. Comparison between HC vs FMD with tremor (without dystonia) vs OMDs Tremor Sub-group for postural control parameters.

	HC Mean ± SD (n =153)	FMD with tremor (without dystonia) (n=50)	OMD - Tremor Mean ± SD (n =44)	p-value post-hoc	Cohen's d
			**Single Task**		
**EO sway area**	166.19 ± 84.61	422.04 ± 352.03	211,10 ± 117,00	**<0,001(1-2; 2-3)**	d_{1-2}= -0.93; d_{2-3} = 0.67
**EO sway perimeter**	257.59 ± 63.99	419.72 ± 240.35	336,01 ± 71,39	**<0,001(1-2;1-3)**	d_{1-2}= -0.88; d_{1-3} = -1.22
**EC sway area**	348.58 ± 226.25	1311.52 ± 1103.95	448,10 ± 279,35	**<0,001(1-2; 2-3)**	d_{1-2}= -1.07; d_{2-3} = 0.84
**EC sway perimeter**	444.98 ± 158.55	790.49 ± 477.05	590,97 ± 248,22	**<0,001(1-2;1-3)**	d_{1-2}= -0.89; d_{1-3} = -0.74
**Romberg index (%)**	205.36± 91.00	279.01 ± 163.57	223,57 ± 105,03	**0.002 (1-2)**	d_{1-2}= -0.58
			**Motor Dual task**		
**EO sway area**	211.45 ± 92.18	495.58 ± 402.87	247,34 ± 139,94	**<0,001(1-2; 2-3)**	d_{1-2}= -0.86; d_{2-3} = 0.75
**EO sway perimeter**	466.90 ± 165.81	720.30 ± 487.10	415,93 ± 112,66	**<0,001(1-2; 2-3)**	d_{1-2}= -0.62; d_{1-3} = 0.85
**EC sway area**	398.55 ± 193.70	929.72 ± 626.66	451,91 ± 279,05	**<0,001(1-2; 2-3)**	d_{1-2}= -1.03; d_{2-3} = 0.86
**EC sway perimeter**	637.40 ± 259.72	968.70 ± 556.47	641,26 ± 206,76	**0.002 (1-2; 2-3)**	d_{1-2}= -0.70; d_{2-3} = -0.69
**Romberg index (%)**	174.48 ± 81.59	191.82 ± 91.02	196,20 ± 101,42	0,539	-
			**Cognitive dual task**		
**EO sway area**	224.95 ± 157.96	504.97 ± 378.52	298,90 ± 145,99	**0.007(1-3), <0.001 (1-2); 0.014 (2-3)**	d_{1-2}= -0.88; d_{1-3} = -0.53; d_{2-3} = 0.65
**EO sway perimeter**	326.31 ± 91.91	464.26 ± 179.44	455,19 ± 147,57	**<0,001(1-2;1-3)**	d_{1-2}= -0.93; d_{1-3} = -0.99
**EC sway area**	285.43 ± 159.22	767.49 ± 673.44	439,47 ± 315,55	**<0,001(1-2;1-3), 0.009 (2-3)**	d_{1-2}= -0.91; d_{2-3} = 0.57; d_{1-3} = -0.72
**EC sway perimeter**	443.93 ± 147.24	642.83 ± 300.79	612,92 ± 223,26	**<0,001(1-2;1-3)**	d_{1-2}= -0.76; d_{1-3}<0.20
**Romberg index (%)**	142.65 ± 92.73	150.75 ± 76.57	155,99 ± 67,73	0,061	-

Table S7 Main interpretation: Gait Analysis performance was comparable in the OMDs group between essential tremor and dystonia subgroups. The analysis showed no significant between-group differences detected in any condition.

Table S7. Comparison between OMDs sub-groups for gait parameters.

	Essential Tremor Mean ± SD	Dystonia Mean ± SD	p-value
	**Single Task**		
Stride length	1,10 ± 0,16	1,11 ± 0,19	0,636
Stride time	1,21 ± 0,12	1,21 ± 0,12	0,681
Speed	0,91 ± 0,17	0,92 ± 0,19	0,848
Stride time var	3,32 ± 1,44	3,10 ± 1,31	0,846
	**Motor Dual Task**		
Stride length	1,09 ± 0,16	1,08 ± 0,17	0,749
Stride time	1,24 ± 0,11	1,25 ± 0,12	0,980
Speed	0,88 ± 0,14	0,88 ± 0,16	0,922
Stride time var	2,99 ± 1,53	2,74 ± 1,31	0,805
	**Cognitive Dual Task**		
Stride length	1,03 ± 0,14	1,05 ± 0,19	0,619
Stride time	1,32 ± 0,19	1,34 ± 0,17	0,806
Speed	0,77 ± 0,18	0,80 ± 0,19	0,788
Stride time var	3,99 ± 2,28	4,08 ± 2,30	0,808
	**Visual Dual Task**		
Stride length	1,12 ± 0,15	1,16 ± 0,16	0,312
Stride time	1,17 ± 0,11	1,20 ± 0,09	0,338
Speed	0,94 ± 0,16	0,94 ± 0,19	0,756
Stride time var	2,89 ± 1,01	2,54 ± 1,21	0,124

Table S8 Main interpretation: Gait analysis performance was comparable between FMDs subgroups (isolated motor manifestations): No significant differences emerged in postural control or gait performance across single-task and dual-task conditions; however, these analyses were conducted on a reduced sample size (<10 subjects per subgroup).

Table S8. Comparison between FMDs sub-groups for gait parameters

	Weaknesses Mean ± SD	Tremor Mean ± SD	Dystonia Mean ± SD	Gait Impairment Mean ± SD	p-value
			**Single task**		
Stride length	1,10 ± 0,26	1,13 ± 0,13	1,18 ± 0,26	1,16 ± 0,09	0,922
Stride time	1,20 ± 0,04	1,21 ± 0,04	1,21 ± 0,15	1,13 ± 0,07	0,163
Speed	0,96 ± 0,20	0,91 ± 0,12	0,98 ± 0,28	0,98 ± 0,10	0,900
Stride time var	3,29 ± 1,22	3,75 ± 1,73	3,89 ± 2,14	1,58 ± 0,57	0,174
			**Motor Dual Task**		
Stride length	1,12 ± 0,22	1,14 ± 0,20	1,11 ± 0,19	1,18 ± 0,07	0,952
Stride time	1,24 ± 0,11	1,26 ± 0,06	1,24 ± 0,16	1,24 ± 0,14	0,585
Speed	0,90 ± 0,24	0,88 ± 0,18	0,91 ± 0,22	0,99 ± 0,09	0,861
Stride time var	3,50 ± 1,79	3,86 ± 1,51	3,28 ± 2,29	2,20 ± 0,16	0,556
			**Cognitive Dual Task**		
Stride length	1,07 ± 0,15	1,07 ± 0,14	1,09 ± 0,22	1,12 ± 0,29	0,966
Stride time	1,50 ± 0,34	1,33 ± 0,15	1,29 ± 0,18	1,20 ± 0,06	0,140
Speed	0,74 ± 0,22	0,80 ± 0,10	0,87 ± 0,22	0,95 ± 0,27	0,386
Stride time var	3,43 ± 1,27	4,78 ± 2,90	3,30 ± 2,17	2,95 ± 1,61	0,600
			**Visual Dual Task**		
Stride length	1,13 ± 0,21	1,18 ± 0,21	1,15 ± 0,24	1,36 ± 0,30	0,783
Stride time	1,20 ± 0,06	1,22 ± 0,07	1,19 ± 0,14	1,13 ± 0,06	0,233
Speed	0,92 ± 0,25	0,96 ± 0,22	0,98 ± 0,26	1,21 ± 0,32	0,701
Stride time var	2,73 ± 1,25	3,86 ± 1,95	2,94 ± 1,06	2,05 ± 0,57	0,314

Table S9 Main interpretation: Gait Analysis performance was comparable among HCs, FMDs, and OMDs with tremor phenotypes: statistical significant differences emerged in postural control across single-task and dual-task conditions.

Table S9. Comparison between HCs vs FMDs vs OMDs Tremor Sub-group for gait analysis parameters.

	**1. HC Mean ± SD (n = 153)**	**2. FMD Mean ± SD (n = 110)**	**3. OMD - Tremor Mean ± SD (n=44)**	**p-value post-hoc**	**Cohen's d**
			**Single Task**		
**Stride Length**	1.27 ± 0.13	1.17 ± 0.27	1,10 ± 0,16	**<0,001(1-2; 2-3;1-3)**	d_{1-2}= 0.47; d_{2-3} = 0.30; d_{1-3} = 1.09
**Gait Cycle Duration**	1.14 ± 0.08	1.20 ± 0.13	1,21 ± 0,12	**<0,001(1-2;1-3)**	d_{1-2}= -0.56; d_{1-3} = -0.71
**Speed**	1.11 ± 0.15	0.96 ± 0.30	0,91 ± 0,17	**<0,001(1-2;1-3)**	d_{1-2}= 0.61; d_{1-3} = 1.28
**Stride Time Variability**	3.00 ± 1.32	3.68 ± 1.79	3,32 ± 1,44	**<0,001(1-2)**	d_{1-2}= -0.43
			**Motor Dual task**		
**Stride Length**	1.25 ± 0.14	1.11 ± 0.28	1,09 ± 0,16	**<0,001(1-2; 1-3)**	d_{1-2}= 0.64; d_{1-3} = 1.19
**Gait Cycle Duration**	1.17 ± 0.10	1.27 ± 0.16	1,24 ± 0,11	**<0,001(1-2; 1-3)**	d_{1-2}= -0.74; d_{1-3} = -0.65
**Speed**	1.09 ± 0.15	0.89 ± 0.30	0,88 ± 0,14	**<0,001(1-2; 1-3)**	d_{1-2}= 0.81; d_{1-3} = 1.42
**Stride Time Variability**	2.76 ± 1.26	4.15 ± 2.14	2,99 ± 1,53	**<0,001(1-2; 2-3)**	d_{1-2}= -0.78; d_{2-3} = 0.61
			**Cognitive dual task**		
**Stride Length**	1.20 ± 0.15	1.07 ± 0.25	1,03 ± 0,14	**<0,001(1-2; 1-3)**	d_{1-2}= 0.61; d_{1-3} = 1.33
**Gait Cycle Duration**	1.23 ± 0.13	1.33 ± 0.20	1,32 ± 0,19	**<0,001(1-2;1-3)**	d_{1-2}= -0.60; d_{1-3} = -0.60
**Speed**	0.98 ± 0.17	0.81 ± 0.27	0,77 ± 0,18	**<0,001(1-2;1-3)**	d_{1-2}= 0.74; d_{1-3} = 1.36
**Stride Time Variability**	3.16 ± 1.42	4.20 ± 3.00	3,99 ± 2,28	**<0,001(1-2;2-3)**	d_{1-2}= -0.44; d_{2-3} <0.20
			**Visual Dual Task**		
**Stride Length**	1.31 ± 0.13	1.15 ± 0.28	1,12 ± 0,15	**<0,001(1-2; 1-3)**	d_{1-2}= 0.73; d_{1-3} = 1.48
**Gait Cycle Duration**	1.11 ± 0.13	1.20 ± 0.13	1,17 ± 0,11	**<0,001(1-2; 1-3)**	d_{1-2}= -0.69; d_{1-3} = -0.51
**Speed**	1.17 ± 0.14	0.96 ± 0.30	0,94 ± 0,16	**<0,001(1-2; 1-3)**	d_{1-2}= 0.87; d_{1-3} = 1.52
**Stride Time Variability**	2.51 ± 1.14	3.75 ± 2.17	2,89 ± 1,01	**<0,001(1-2)**	d_{1-2}= -0.72

Table S10 Main interpretation: Gait Analysis performance was comparable among HCs, FMDs, and OMDs with dystonia phenotypes: statistically significant differences emerged in postural control across single-task and dual-task conditions.

Table S10. Comparison between HCs vs FMDs vs OMDs Dystonia Sub-group for gait analysis parameters.

	**1. HC Mean ± SD (n = 153)**	**2. FMD Mean ± SD (n = 110)**	**3.OMD - Dystonia Mean ± SD (n = 52)**	**p-value post-hoc**	**Cohen's d**
			**Single Task**		
**Stride Length**	1.27 ± 0.13	1.17 ± 0.27	1,11 ± 0,19	**<0,001(1-2;1-3)**	d_{1-2}= 0.47; d_{1-3} = 1.01

**Gait Cycle Duration**	1.14 ± 0.08	1.20 ± 0.13	1,21 ± 0,12	**<0,001(1-2;1-3)**	d_{1-2}= -0.56; d_{1-3} = -0.71
**Speed**	1.11 ± 0.15	0.96 ± 0.30	0,92 ± 0,19	**<0,001(1-2;1-3)**	d_{1-2}= 0.61; d_{1-3} = 1.10
**Stride Time Variability**	3.00 ± 1.32	3.68 ± 1.79	3,10 ± 1,31	**<0,001(1-2;2-3)**	d_{1-2}= -0.43; d_{2-3}= 0.40
			**Motor Dual task**		
**Stride Length**	1.25 ± 0.14	1.11 ± 0.28	1,08 ± 0,17	**<0,001(1-2; 1-3)**	d_{1-2}= 0.64; d_{1-3} = 1.17
**Gait Cycle Duration**	1.17 ± 0.10	1.27 ± 0.16	1,25 ± 0,12	**<0,001(1-2; 1-3)**	d_{1-2}= -0.74; d_{1-3} = -0.65
**Speed**	1.09 ± 0.15	0.89 ± 0.30	0,88 ± 0,16	**<0,001(1-2; 1-3)**	d_{1-2}= 0.81; d_{1-3} = 1.39
**Stride Time Variability**	2.76 ± 1.26	4.15 ± 2.14	2,74 ± 1,31	**<0,001(1-2; 2-3)**	d_{1-2}= -0.78; d_{2-3}= 0.75
			**Cognitive dual task**		
**Stride Length**	1.20 ± 0.15	1.07 ± 0.25	1,05 ± 0,19	**<0,001(1-2; 1-3)**	d_{1-2}= 0.64; d_{1-3} = 1.17
**Gait Cycle Duration**	1.23 ± 0.13	1.33 ± 0.20	1,34 ± 0,17	**<0,001(1-2;1-3)**	d_{1-2}= -0.74; d_{1-3} = -0.65
**Speed**	0.98 ± 0.17	0.81 ± 0.27	0,80 ± 0,19	**<0,001(1-2;1-3)**	d_{1-2}= 0.81; d_{1-3} = 1.39
**Stride Time Variability**	3.16 ± 1.42	4.20 ± 3.00	4,08 ± 2,30	**<0,001(1-2)**	d_{1-2}= -0.78; d_{2-3}= 0.75
			**Visual Dual Task**		
**Stride Length**	1.31 ± 0.13	1.15 ± 0.28	1,16 ± 0,16	**<0,001(1-2; 1-3)**	d_{1-2}= 0.61; d_{1-3} = 1.20
**Gait Cycle Duration**	1.11 ± 0.13	1.20 ± 0.13	1,20 ± 0,09	**<0,001(1-2; 1-3)**	d_{1-2}= -0.60; d_{1-3} = -0.67
**Speed**	1.17 ± 0.14	0.96 ± 0.30	0,94 ± 0,19	**<0,001(1-2; 1-3)**	d_{1-2}= 0.74; d_{1-3} = 1.30
**Stride Time Variability**	2.51 ± 1.14	3.75 ± 2.17	2,54 ± 1,21	**<0,001(1-2;2-3)**	d_{1-2}= -0.43; d_{2-3}<0.20

Table S11 Main interpretation: Gait Analysis performance was compared among HCs, FMD patients presenting tremor symptoms (including patients with additional motor manifestations but without dystonia), and OMD patients with tremor manifestations.

Table S11. Comparison between HC vs FMD vs OMDs Tremor Sub-group for gait analysis parameters.

	**HC Mean ± SD (n =153)**	**FMD with tremor (without dystonia) (n=50))**	**OMD - Tremor Mean ± SD (n =44)**	**p-value post-hoc**	**Cohen's d**
			**Single Task**		
**Stride Length**	1.27 ± 0.13	1.18 ± 0.22	1,10 ± 0,16	**<0.001(1-2; 1-3), 0.035 (2-3)**	d_{1-2}= 0.49; d_{1-3} = 1.23; d_{2-3}= 0.40
**Gait Cycle Duration**	1.14 ± 0.08	1.19 ± 0.09	1,21 ± 0,12	**<0.001(1-2;1-3)**	d_{1-2}= -0.63; d_{1-3} = -0.83
**Speed**	1.11 ± 0.15	0.96 ± 0.26	0,91 ± 0,17	**<0.001(1-2;1-3)**	d_{1-2}= 0.62; d_{1-3} = 1.25
**Stride Time Variability**	3.00 ± 1.32	3.70 ± 1.72	3,32 ± 1,44	**<0.001(1-2)**	d_{1-2}= -0.45
			**Motor Dual task**		
**Stride Length**	1.25 ± 0.14	1.10 ± 0.22	1,09 ± 0,16	**<0.001(1-2; 1-3)**	d_{1-2}= 0.78; d_{1-3} = 1.13
**Gait Cycle Duration**	1.17 ± 0.10	1.27 ± 0.14	1,24 ± 0,11	**<0.001(1-2; 1-3)**	d_{1-2}= -0.83; d_{1-3} = -0.67
**Speed**	1.09 ± 0.15	0.88 ± 0.26	0,88 ± 0,14	**<0.001(1-2; 1-3)**	d_{1-2}= 0.98; d_{1-3} = 1.38
**Stride Time Variability**	2.76 ± 1.26	4.73 ± 2.82	2,99 ± 1,53	**<0.001(1-2; 2-3)**	d_{1-2}= -0.85; d_{2-3}=0.74
			**Cognitive dual task**		
**Stride Length**	1.20 ± 0.15	1.09 ± 0.21	1,03 ± 0,14	**<0.001(1-2; 1-3), 0.020 (2-3)**	d_{1-2}= 0.63; d_{1-3} = 1.17; d_{2-3}= 0.40
**Gait Cycle Duration**	1.23 ± 0.13	1.34 ± 0.18	1,32 ± 0,19	**0.005(1-2;1-3)**	d_{1-2}= -0.71; d_{1-3} = -0.53
**Speed**	0.98 ± 0.17	0.80 ± 0.22	0,77 ± 0,18	**<0.001(1-2;1-3)**	d_{1-2}= 0.95; d_{1-3} = 1.23
**Stride Time Variability**	3.16 ± 1.42	4.60 ± 1.88	3,99 ± 2,28	**<0.001(1-2), 0.013 (2-3)**	d_{1-2}= -0.83; d_{2-3}=0.35; $d1_{-3}$= -0.40
			**Visual Dual Task**		
**Stride Length**	1.31 ± 0.13	1.16 ± 0.24	1,12 ± 0,15	**<0,001(1-2; 1-3)**	d_{1-2}= 0.74; d_{1-3} = 1.36
**Gait Cycle Duration**	1.11 ± 0.13	1.20 ± 0.12	1,17 ± 0,11	**<0,001(1-2; 1-3)**	d_{1-2}= -0.72; d_{1-3} = -0.49
**Speed**	1.17 ± 0.14	0.96 ± 0.26	0,94 ± 0,16	**<0,001(1-2; 1-3)**	d_{1-2}= 0.96; d_{1-3} = 1.54
**Stride Time Variability**	2.51 ± 1.14	3.92 ± 2.05	2,89 ± 1,01	**<0,001(1-2)**	d_{1-2}= -0.81

Table S12 Main interpretation: Group comparisons revealed a consistent reduction in cognitive output in FMD and OMD patients compared to HC during dual-task postural conditions, while accuracy (error rates) was largely preserved, indicating that group differences were mainly driven by reduced cognitive throughput rather than impaired accuracy. In FMDs, cognitive performance remained stable across EO and EC conditions, suggesting limited modulation by visual input and no cognitive cost associated with postural improvements under EC conditions. During gait, an increase in error rates was observed only in OMD patients, indicating greater vulnerability of cognitive performance under dynamic motor conditions in this group.

Table S12. Comparison on cognitive output (mean number of responses) and mean percentage of errors for each experimental condition among groups.

**Variables**	**HC**	**FMD**	**OMD**	**p-value Between Groups**

**Postural OA Responses (n)**	8.20	4.78	4.96	< 0.001 (HC vs FMD); 0.004 (HC vs OMD)
**Postural OA Errors (%)**	13.05%	32.05%	28.23%	0.754
**Postural OC Responses (n)**	8.29	5.02	4.51	< 0.001 (HC vs FMD; HC vs OMD)
**Postural OC Errors (%)**	13.63%	31.08%	33.48%	0.639
**p-value Within-group**	0.354	0.542	0.695	
**Variables**	**HC**	**FMD**	**OMD**	**p-value**
**Gait Responses (n)**	3.83	3.27	2.78	0.089
**Gait Errors (%)**	21.15%	31.50%	50.72%	0.038 (HC vs OMD)

Abbreviations: OA, eyes open; OC, eyes closed; HC, healthy controls; FMD, functional movement disorders; OMD, organic movement disorders.

Table 13 Main interpretation: Comparison analysis between FMDs and HCs using a height-matched sample to account for the potential confounding effect of anthropometric differences.

Table S13. Between-group comparisons using the Mann–Whitney U test. Significant differences are reported in bold.

Variables	HC group n = 68		FMD group n = 99		p-value
age	43,721	14,587	44,111	13,871	0.861
height	1,644	0,042	1,687	0,077	0,052
SINGLE TASK					
Stride length	1,265	0,107	1,188	0,255	**<0,001**
Stride time	1,133	0,085	1,205	0,128	**<0,001**
Speed	1,115	0,130	0,956	0,293	**<0,001**
Stride time var	3,012	1,015	3,736	2,033	**<0,001**
MOTOR DUAL TASK					
Stride length	1,219	0,118	1,116	0,262	**<0,001**
Stride time	1,167	0,098	1,278	0,158	**<0,001**
Speed	1,061	0,131	0,886	0,293	**<0,001**
Stride time var	2,798	1,140	4,514	2,597	**<0,001**
COGNITIVE DUAL TASK					
Stride length	1,181	0,127	1,099	0,232	**<0,001**
Stride time	1,233	0,130	1,340	0,203	**<0,001**
Speed	0,959	0,157	0,811	0,256	**<0,001**
Stride time var	3,209	1,316	4,736	3,526	**<0,001**
VISUAL DUAL TASK					
Stride length	1,291	0,101	1,159	0,271	**<0,001**
Stride time	1,113	0,076	1,193	0,120	**<0,001**
Speed	1,169	0,130	0,956	0,295	**<0,001**
Stride time var	2,509	1,030	3,908	2,431	**<0,001**

Table S14 Main interpretation: Comparison analysis between FMDs and HCs using a gait speed-matched sample to account for the potential confounding effect of gait speed. Participants were matched on gait speed, while the groups remained comparable for the other demographic and anthropometric variables considered in the primary analyses (age, gender, height, and education).

Table S14. Gait parameters in HCs and FMDs matched for gait speed.

Variable	HCs n = 68	FMDs n = 70	p-value
**Single Task**			
Speed	1.11 ± 0.13	1.09 ± 0.22	0.405
Stride length	1.27 ± 0.11	1.30 ± 0.19	0.151
Stride time	1.13 ± 0.09	1.18 ± 0.11	0.172
Stride time var	3.01 ± 1.02	3.24 ± 1.77	0.202
**Motor Dual Task**			
Speed	1.06 ± 0.13	1.02 ± 0.22	0.402
Stride length	1.22 ± 0.12	1.23 ± 0.18	**0.005**
Stride time	1.17 ± 0.10	1.24 ± 0.15	**0.004**
Stride time var	2.80 ± 1.14	3.88 ± 2.09	**< 0,001**
**Cognitive Dual Task**			
Speed	0.96 ± 0.16	0.92 ± 0.19	0.153
Stride length	1.18 ± 0.13	1.20 ± 0.15	0.346
Stride time	1.23 ± 0.13	1.30 ± 0.15	**< 0,001**
Stride time var	3.21 ± 1.32	4.41 ± 3.59	0.677
**Visual Dual Task**			
Speed	1.12 ± 0.13	1.10 ± 0.25	0,160
Stride length	1.29 ± 0.10	1.26 ± 0.22	**0.001**
Stride time	1.11 ± 0.08	1.18 ± 0.12	**< 0,001**
Stride time var	2.51 ± 1.03	3.35 ± 2.20	**< 0,001**

STROBE Statement—Checklist of items that should be included in reports of ***cross-sectional studies***

	Item No	Recommendation	Section
**Title and abstract**	1	(*a*) Indicate the study's design with a commonly used term in the title or the abstract	Title
		(*b*) Provide in the abstract an informative and balanced summary of what was done and what was found	Abstract
**Introduction**			
Background/rationale	2	Explain the scientific background and rationale for the investigation being reported	Introduction
Objectives	3	State specific objectives, including any prespecified hypotheses	Introduction
**Methods**			
Study design	4	Present key elements of study design early in the paper	Materials and methods
Setting	5	Describe the setting, locations, and relevant dates, including periods of recruitment, exposure, follow-up, and data collection	Materials and methods
Participants	6	(*a*) Give the eligibility criteria, and the sources and methods of selection of participants	Materials and methods
Variables	7	Clearly define all outcomes, exposures, predictors, potential confounders, and effect modifiers. Give diagnostic criteria, if applicable	Materials and methods
Data sources/ measurement	8*	For each variable of interest, give sources of data and details of methods of assessment (measurement). Describe comparability of assessment methods if there is more than one group	Materials and methods
Bias	9	Describe any efforts to address potential sources of bias	Materials and methods
Study size	10	Explain how the study size was arrived at	Materials and methods
Quantitative variables	11	Explain how quantitative variables were handled in the analyses. If applicable, describe which groupings were chosen and why	Materials and methods
Statistical methods	12	(*a*) Describe all statistical methods, including those used to control for confounding	Materials and methods
		(*b*) Describe any methods used to examine subgroups and interactions	Materials and methods
		(*c*) Explain how missing data were addressed	Materials and methods
		(*d*) If applicable, describe analytical methods taking account of sampling strategy	n.a.

		(*e*) Describe any sensitivity analyses	Materials and methods, Suppl Tables S2-S11
**Results**			
Participants	13*	(a) Report numbers of individuals at each stage of study—eg numbers potentially eligible, examined for eligibility, confirmed eligible, included in the study, completing follow-up, and analysed	Results, Figure 1
		(b) Give reasons for non-participation at each stage	Results, Figure 1
		(c) Consider use of a flow diagram	Figure 1
Descriptive data	14*	(a) Give characteristics of study participants (eg demographic, clinical, social) and information on exposures and potential confounders	Results, Table 1, Supplementary Table S1
		(b) Indicate number of participants with missing data for each variable of interest	Results
Outcome data	15*	Report numbers of outcome events or summary measures	Results, Table 2-6
Main results	16	(*a*) Give unadjusted estimates and, if applicable, confounder-adjusted estimates and their precision (eg, 95% confidence interval). Make clear which confounders were adjusted for and why they were included	Results, Tables 2-6
		(*b*) Report category boundaries when continuous variables were categorized	n.a.
		(*c*) If relevant, consider translating estimates of relative risk into absolute risk for a meaningful time period	n.a.
Other analyses	17	Report other analyses done—eg analyses of subgroups and interactions, and sensitivity analyses	Results, Supplementary Tables S2-S14
**Discussion**			
Key results	18	Summarise key results with reference to study objectives	Discussion
Limitations	19	Discuss limitations of the study, taking into account sources of potential bias or imprecision. Discuss both direction and magnitude of any potential bias	Discussion
Interpretation	20	Give a cautious overall interpretation of results considering objectives, limitations, multiplicity of analyses, results from similar studies, and other relevant evidence	Discussion
Generalisability	21	Discuss the generalisability (external validity) of the study results	Discussion
**Other information**			
Funding	22	Give the source of funding and the role of the funders for the present study and, if applicable, for the original study on which the present article is based	Funding

*Give information separately for exposed and unexposed groups.

Note: An Explanation and Elaboration article discusses each checklist item and gives methodological background and published examples of transparent reporting. The STROBE checklist is best used in conjunction with this article (freely available on the Web sites of PLoS Medicine at http://www.plosmedicine.org/, Annals of Internal Medicine at http://www.annals.org/, and Epidemiology at http://www.epidem.com/). Information on the STROBE Initiative is available at www.strobe-statement.org.
